# Dissolving Microneedles with Spatiotemporally controlled pulsatile release Nanosystem for Synergistic Chemo-photothermal Therapy of Melanoma

**DOI:** 10.7150/thno.44194

**Published:** 2020-07-09

**Authors:** Wanbing Qin, Guilan Quan, Ying Sun, Minglong Chen, Peipei Yang, Disang Feng, Ting Wen, Xinyu Hu, Xin Pan, Chuanbin Wu

**Affiliations:** 1 School of Pharmaceutical Sciences, Sun Yat-sen University, Guangzhou 510006, China.; 2 College of Pharmacy, Jinan University, Guangzhou, 510632, China.; 3 Department of Pharmacy, Guangzhou Women and Children's Medical Center, Guangzhou Medical University, Guangzhou 510623, China.

**Keywords:** melanoma, dissolving microneedles, solid lipid nanoparticles, triggered release, chemo-photothermal therapy

## Abstract

High aggressiveness and recurrence of melanoma tumors require multiple systemic drug administrations, causing discomfort and severe side effects to the patients. Topical treatment strategies that provide repetitively controllable and precise drug administrations will greatly improve treatment effects.

**Methods:** In this study, a spatiotemporally controlled pulsatile release system, which combined dissolving microneedles (DMNs) and thermal-sensitive solid lipid nanoparticles (SLNs), was constructed to realize multiple doses of dual-modal chemo-photothermal therapy in a single administration. Paclitaxel (PTX) and photothermal agent IR-780 were encapsulated into SLNs and were concentrated in the tips of DMNs (PTX/IR-780 SLNs @DMNs). Equipped with several needles, the DMN patch could be directly inserted into the tumor site and provide a stable “Zone accumulation” to constrain the PTX/IR-780 SLNs at the tumor site with uniform distribution.

**Results:** *In vitro* experiments showed that after irradiation with near-infrared light, the PTX/IR-780 SLNs gradually underwent phase transition, thereby accelerating the release of PTX. When irradiation was switched off, the PTX/IR-780 SLNs cooled to re-solidify with limited drug release. Compared with intravenous and intratumoral injections, very few SLNs from PTX/IR-780 SLNs @DMNs were distributed into other organs, resulting in enhanced bioavailability at the tumor site and good safety. *In vivo* analysis revealed that PTX/IR-780 SLNs @DMNs exhibited significant anti-tumor efficacy. In particular, the primary tumor was completely eradicated with a curable rate of 100% in 30 days and the highest survival rate of 66.67% after 100 days of treatment.

**Conclusion:** Herein, we developed a DMN system with a unique spatiotemporally controlled pulsatile release feature that provides a user-friendly and low-toxicity treatment route for patients who need long-term and repeat treatments.

## Introduction

Skin cancers represent a growing proportion of all human cancers and are associated with high mortality 1. Although it only accounts for 4% of all skin cancer cases, malignant melanoma is the main cause of death in superficial skin tumors 2, 3. Surgery and chemotherapy are the major treatments for malignant melanoma. However, there is a risk of tumor recurrence if the tumor tissue is not completely removed. Also, patients with some underlying diseases are intolerant to surgery. Traditional chemotherapy shows unsatisfactory efficacy in malignant melanoma and toxic side effects usually lead to poor quality of life 4. Another option proposed for the treatment of melanoma was of external microenergy, including ultrasound therapy 5-7, magnetic response therapy 8, 9, and photothermal therapy (PTT) 10-12. PTT can induce thermal ablation of tumor cells by converting light energy to heat using photothermal agents, causing irreversible damage to tumors but no damage to normal tissues. Therefore, PTT represents a potential noninvasive strategy for superficial skin tumor therapy. In recent years, the combination of chemotherapy and PTT has exhibited synergetic therapeutic effects with low dose requirements, therefore, inspired research in this area 12-15.

There are several photothermal agents, such as gold nanoparticles 16, 17, carbon nanotubes 18, 19, graphene oxide 20, 21, and near-infrared (NIR) dye 22, 23. Among these, NIR dye has optimal absorption radiation at 700 nm - 900 nm, which is a transparent window for organisms, and is widely used in medical imaging and photothermal ablation therapy. IR-780 is a typical NIR dye, which shows strong photostability and improved photothermal effects 24-26. The poorly water-soluble IR-780 is quite unstable in aqueous medium and is quickly cleared from the body.

With recent advances in nanotechnology research, nanocarriers have shown great potential to solubilize poorly insoluble agents and stabilize sensitive cargos. Examples of such nanocarriers include mesoporous silica nanoparticles 27-29, metal-organic framework 30, 31, micelles 15, 32, and solid lipid nanoparticles (SLNs) 33. In recent years, the development of temperature-sensitive nanoparticles represents a new strategy for the controlled release of drugs 34, 35. These nanodrug delivery systems are commonly administered by intravenous injections. However, long-term systemic circulation and undesirable drug leakage severely impede effective drug accumulation at the tumor site and cause inevitable side effects to healthy organs. Also, frequent multiple doses are often needed to better control the growth of tumors, resulting in patient discomfort and damage. Hence, the development of an ideal topical delivery strategy with repeated dosing in a single administration is critical for tumors, such as superficial melanoma, to enhance drug accumulation at the lesion location, reduce systemic toxic effects, and decrease administration frequency.

Dissolving microneedles (DMNs) are micrometer length needle arrays, which create hundreds of micro-pathways in the skin via direct insertion into the stratum corneum in a minimally invasive manner with low pain and improved transdermal drug delivery efficacy 36, 37. In this context, DMNs are suitable for effectively accumulating nanodrug-loaded systems at the lesion site of many superficial tumors 38-41. After administration, spatially controlled drug distribution and permeation are realized and the well-designed needle tips can be dissolved by the tissue fluid and gradually release the encapsulated nanocarriers. Furthermore, side effects in normal tissues and organs associated with systemic circulation can be remarkably reduced. The development of emerging microneedle manufacturing methods would enable more flexibility in the design of individualized microneedles according to the characteristics of the lesion site 42-44. Also, various materials can be selected to prepare functionalized microneedles. For example, Chen et al. developed a light-activatable polycaprolactone microneedle system to repeatedly trigger small molecular drug release, providing a user-friendly and low toxicity option for patients who need long-term treatment 45-47.

**Figure 1:**
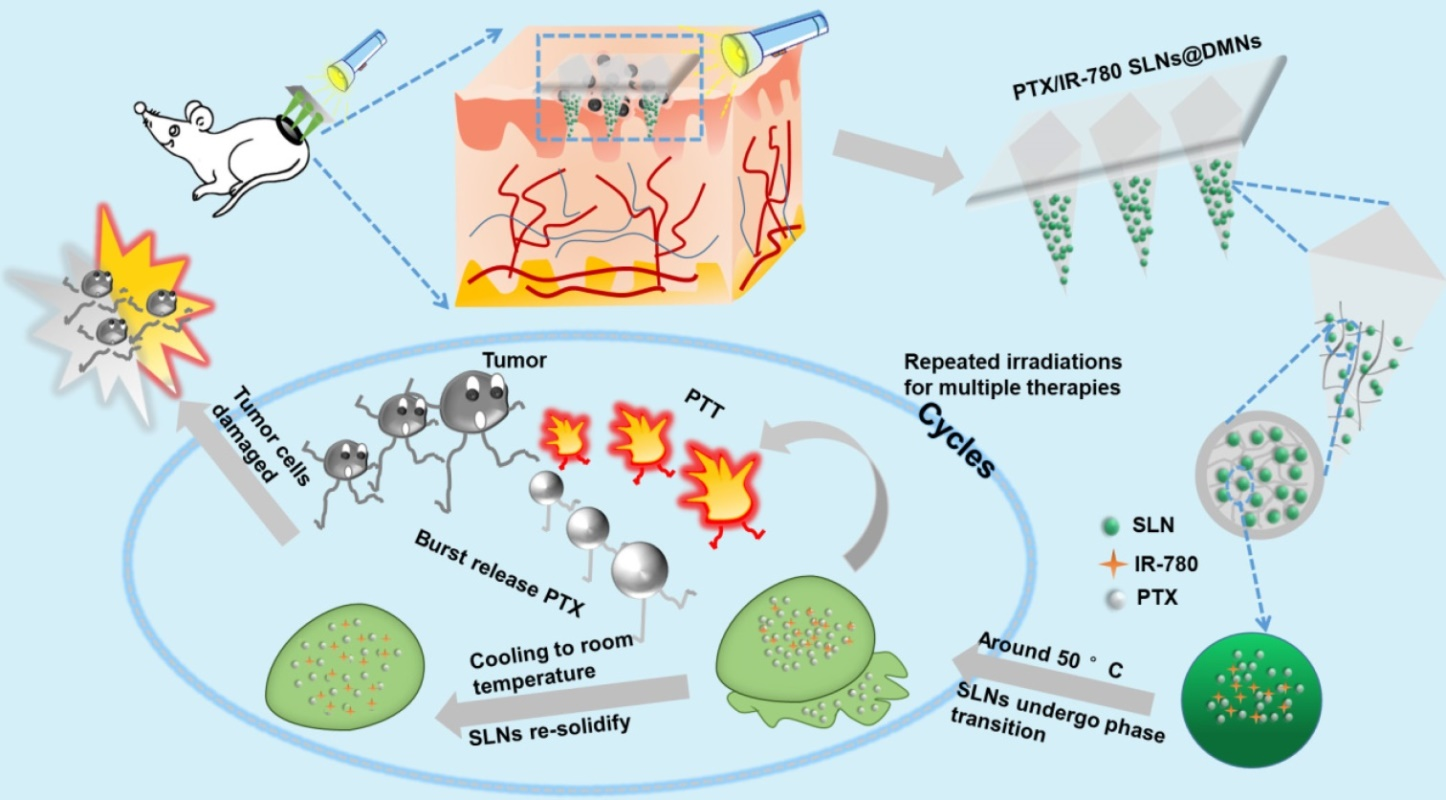
Schematic illustration of spatiotemporally controlled pulsatile release DMNs drug delivery system for melanoma therapy.

The purpose of this study was to develop nanocarrier-packaged DMNs for effective melanoma therapy. The chemotherapy drug paclitaxel (PTX) and photothermal agent IR-780 were co-loaded into thermal-sensitive SLNs to achieve temporally controlled multiple doses in a single administration (**Figure 1**). The SLNs were concentrated at the needle tips of DMNs for accurate delivery. After direct insertion into the tumor site, PTX/IR-780 SLNs were released and were uniformly distributed at the site. When irradiation was applied to the tumor site, IR-780 absorbed light energy and converted it into heat, leading to an *in situ* phase transition of SLNs followed by PTX burst release. Once the laser was switched off, the temperature decreased, SLNs were re-solidified, and the PTX release was limited. In subsequent analyses, the pharmacodynamics of the formulations were evaluated *in vitro* and *in vivo*. Thus, the spatiotemporally controlled PTX/IR-780 SLNs @DMNs developed in this study provided multiple chemo-photothermal therapies in a single administration and exhibited obvious superiority in inhibiting tumor growth compared with intravenous and intratumoral injections of PTX/IR-780 SLNs.

## Methods

### Materials

Tricaprin (TA) was purchased from Tokyo Chemical Industry Co., Ltd. (Tokyo, Japan). Cetyl Palmitate (CP), PTX, and gelatin were purchased from Aladdin (Shanghai, China). IR-780 was obtained from J&K Scientific (Beijing, China). Sodium hyaluronic acid (HA) (MW < 10 kDa) was acquired from Bloomage Freda Biopharm Co., Ltd. (Shanxi, China). Polyvinyl pyrrolidone (PVP K90) was kindly provided by MBCHEM Co (New Jersey, USA). Polydimethylsiloxane (PDMS, Sylgard 184 Silicone Elastomer Kit) was customized by Dow Corning Co. (Michigan, USA). RPMI Medium 1640 basic (Gibco™), fetal bovine serum (FBS) (Gibco™), and trypsin (Gibco™) were purchased from Thermo Fisher Scientific Co., Ltd. (Massachusetts, USA). Cell Counting Kit-8 (CCK-8) was purchased from Dojindo Molecular Technologies, Inc. (Kyushu, Japan). Annexin V-fluorescein isothiocyanate (FITC)/PI (Annexin V-FITC/PI) was obtained from MultiSciences Biotech. Co., Ltd. (Hangzhou, China).

### Animals

All animal experiments were carried out according to the Animal Ethical and Welfare Committee of Sun Yat-sen University protocol (Approval No. SYSU-IACUC-2019-000194) and the National Institutes of Health guidelines for the care and use of laboratory animals. C57 female mice were purchased from Guangdong Medical Experimental Animal Center, and all mice were housed in the Experimental Animal Center of Sun Yat-sen University (Guangdong, China).

### Preparation of PTX/IR-780 SLNs

PTX/IR-780 SLNs were fabricated using a modified ultrasound method as described previously 48. Briefly, 100 mg of CP and 20 mg of TA were mixed to form the lipid phase at 70 °C. The aqueous phase containing Pluronic F-68 in 5 mL of ultrapure water (0.4%, w/v) was heated to the same temperature. When the lipid materials were completely melted, 200 μL of PTX and IR-780 ethanol solution (PTX: 0.200 mg/mL, IR-780: 0.0625 mg/mL) was added into the lipid phase and was heated until a uniform phase formed. Subsequently, the lipid phase was gradually added into the aqueous phase under constant stirring (800 rpm) at 70 °C for 15 min to form a pre-emulsion. Finally, the pre-emulsion was sonicated using Ultrasonic Cell Disrupter (BILON-650Y) with an amplitude of 30% for 2 min in an ice bath, and PTX/IR-780 SLNs were obtained after cooling in the ice bath. PTX SLNs, IR-780 SLNs, and blank SLNs were also prepared as controls according to the same procedure.

### Characterization of PTX/IR-780 SLNs

The dried samples, including CP, TA, and PTX/IR-780 SLNs, containing different ratios of CP to TA (CP : TA = 5 : 1, 3 : 1, and 1 : 1), were analyzed by differential scanning calorimetry (DSC, DSC 200 F3 Maia®, Netzsch, Germany) to detect the samples' melting points. The mean diameter, size distribution, and zeta potential of PTX/IR-780 SLNs were measured by using dynamic light scattering (DLS, Zetasizer Nano, Malvern Instrument, UK). The stability of PTX/IR-780 SLNs was evaluated by measuring the particle size change when stored at 4 °C for 10 days. The particle morphology of PTX/IR-780 SLNs was characterized using transmission electron microscopy (TEM, JEM1400, Hitachi, Japan). To observe the drug distribution in SLNs, coumarin-6 (C6) replaced PTX and was encapsulated together with IR-780 into the SLNs to obtain the fluorescence-labeled nanoparticles; confocal laser scanning microscopy (CLSM, FV3000, Olympus, Japan) was used to capture the fluorescence of SLNs before and after laser irradiation. A fluorescence spectrometer was used to record the emission wavelength of free IR-780 and IR-780 SLNs under an excitation wavelength of 763 nm. The free drug in the SLN suspensions was separated by an ultrafiltration method using centrifugal filter tubes (Millipore, molecular weight cutoff 30 kDa). High-performance liquid chromatography (HPLC, LC-20AT, Shimadzu, Japan) with a mobile phase composed of acetonitrile and water at 53: 47 (v/v) was applied to measure the concentration of PTX. For IR-780, the concentration was measured using fluorescence spectrometry. The encapsulation efficiency (EE) and drug loading (DL) of PTX and IR-780 were determined by the following equations:

EE (%) = [weight of PTX (IR-780) in SLNs/weight of total added PTX (IR 780)] × 100% (1)

DL (%) = [weight of PTX (IR-780) in SLNs/weight of total SLNs] × 100% (2)

### Photothermal property of PTX/IR-780 SLN suspension

To compare the photothermal efficiency of IR-780 solution and PTX/IR-780 SLN suspension, 1 mL of samples with different IR-780 concentrations (20 μg/mL and 60 μg/mL) was added into 24-well plates and exposed to an 808 nm laser (1 W/cm^2^) for 5 min, and the temperature changes were recorded every minute. Different laser powers were used to examine the influence on the photothermal effect. To further evaluate the influence of repeated irradiation on photothermal efficiency, the samples were irradiated for several “ON/OFF” cycles under a 1 W/cm^2^ laser. The IR-780 solution and PTX/IR-780 SLN suspension were placed in light for 1 day to evaluate the stability by detecting their fluorescence.

### Temperature-dependent and laser-triggered drug release

To investigate the thermo-responsive drug release profile *in vitro*, PTX/IR-780 SLNs equivalent to 100 μg of PTX were dispersed in 14 mL of release medium (phosphate-buffered saline (PBS) containing 1% polyoxyethylated castor oil) and equally packed into 9 centrifuge tubes. The tubes were placed in a 50 °C water bath for several “ON/OFF” cycles, then the release medium was collected and replaced with fresh medium at the specified time intervals. The concentration of PTX in the medium was analyzed by HPLC.

To further investigate the effect of laser irradiation on the release of PTX, PTX/IR-780 SLNs equivalent to 63 μg of PTX were dispersed in 9 mL of release medium (PBS containing 1% polyoxyethylated castor oil). After irradiation with an 808 nm laser (1 W/cm^2^) for 5 min, the PTX/IR-780 SLNs suspension was divided equally into 6 centrifuge tubes and then placed in a 37 °C shaker. The PTX concentration was measured at predetermined time points. The PTX/IR-780 SLN suspension without laser irradiation was used as the control.

### Cells culture and *in vitro* cellular uptake

Murine melanoma B16 cells were obtained from the Laboratory Animal Center of Sun Yat‑sen University (from American Type Culture Collection) and cultured in RPMI-1640 medium, supplemented with 10% FBS and 1% penicillin/streptomycin at 37 °C with 5% CO_2_.

B16 cells were seeded in 24-well plates at a density of 1 × 10^5^ cells per well and were cultured overnight. For the *in vitro* cellular uptake study, the C6/IR-780 SLNs were added (IR-780:10 μg/mL, C6: 1 μg/mL). After 2 h of incubation, the cells were washed twice with PBS, fixed with 4% paraformaldehyde for 10 min, and then washed prior to nuclear staining with 4', 6-diamidino-2-phenylindole (DAPI). Finally, the cells were imaged using CLSM.

### Laser- triggered intracellular drug release

B16 cells were seeded in 24-well plates at a density of 1 × 10^5^ cells per well. After overnight culture, the medium was replaced with fresh medium containing C6/IR-780 SLNs and the cells were incubated for another 1 h. The cells were washed with PBS three times, fixed with 4% paraformaldehyde for 10 min, stained with DAPI, and then irradiated with a laser at 808 nm for 5 min (0.5 W/cm^2^ or 1 W/cm^2^). The fluorescence intensity of C6 in the cells before and after irradiation was captured by CLSM.

### *In vitro* cytotoxicity and apoptosis assay

B16 cells were seeded in 96-well plates at a density of 5 × 10^3^ cells per well. After overnight culture, the medium was removed, and fresh medium containing different concentrations of samples (blank SLNs, PTX SLNs, IR-780 SLNs, PTX/IR-780 SLNs, and free PTX/IR-780 solution) was added. Following 4 h of coincubation, the medium was replaced with fresh medium. For the groups containing IR-780, cells were exposed to 808 nm laser with 1 W/cm^2^ for 5 min. After incubation for another 24 h, the CCK-8 kit was used and the absorption of each well was determined by a microplate reader (ELx800, Biotek, USA) to determine the viability of cells. To study the survival rate of cells under two laser irradiation cycles, after the first laser irradiation and 24 h incubation, the cells were irradiated with an 808 nm laser again and incubated for another 24 h. The cells were incubated for 48 h and the CCK-8 assay was used to analyze the viability of cells. The laser irradiation alone group was used as a control.

Cell apoptosis was assessed after treatment with 5.00 μg/mL PTX and 3.33 μg/mL IR-780. Briefly, B16 cells were seeded in 6-well plates at a density of 2 × 10^5^ cells per well and incubated overnight to allow adherence. The medium was removed and replaced with fresh medium containing blank SLNs, PTX SLNs, IR-780 SLNs, PTX/IR-780 SLNs, or free PTX/IR-780 solution for a 4 h incubation. Subsequently, the medium was replaced with fresh medium and the samples containing IR-780 were irradiated with an 808 nm laser with 1 W/cm^2^ for 5 min. The cells were incubated for another 24 h, stained with 5 μL of Annexin V-FITC and 10 μL of PI for 5 min, followed by flow cytometry (EPICS, Beckman Coulter, USA) to detect apoptosis.

### ROS generation detection

Intracellular ROS generation was detected using the DCFH-DA probe. After B16 cells were seeded in 24-well plates at a density of 1 × 10^5^ cells per well and incubated overnight, the medium was replaced with free PTX/IR-780 solution and PTX/IR-780 SLNs (PTX: 5.00 μg/mL, IR-780: 3.33 μg/mL) for a 4 h incubation. After removal of the medium, the DCFH-DA probe was added to the cells and incubated for 20 min. Next, the DCFH-DA probe was replaced with PBS and the cells were irradiated with an 808 nm NIR laser for 5 min. Finally, the cells were stained with Hoechst for 10 min. The cells were washed with PBS and then observed by CLSM.

### Formulation and Characterization of PTX/IR-780 SLNs @DMNs

DMNs were prepared through a three-step centrifugation method as described previously 49. The male mold was made from brass and consisted of 144 needles with a height of 800 μm, a base diameter of 300 μm, and a tip-to-tip space of 750 μm. Subsequently, 12 g of PDMS containing 10% fixative was poured onto the brass male and dried for 1.5 h at 80 °C to obtain a female mold. After the female mold was peeled off, PTX/IR-780 SLN suspension was filled into the female mold under centrifugation at 4000 rpm for 5 min at 4 °C, excess PTX/IR-780 SLN suspension was removed, and the female mold was dried overnight at room temperature in a dryer. Since it was difficult to fill SLNs with low density into the female mold microholes, the SLN suspension was added and centrifuged three times to concentrate the SLNs into the needle tips of DMNs. Next, HA solution (400 mg/mL) was filled into the female mold under centrifugation at 4000 rpm for 5 min at 4 °C. Finally, PVP k90 ethanol solution (312.5 g/L) was poured on the female mold and centrifuged at 4000 rpm and 4 °C to form the base part. After drying at room temperature for 12 h, the PTX/IR-780 SLNs @DMNs were gently peeled off. The PTX SLNs @DMNs and IR-780 SLNs @DMNs were also obtained using the same procedure.

The PTX/IR-780 SLNs @DMNs were observed with a fluorescent stereomicroscope (MZFLIII, LEICA, Germany), CLSM, and scanning electron microscope (SEM, JSM-6330F, Jeol, Japan). To investigate the stability of SLNs after they were loaded into DMNs, the particle size of SLNs released from DMNs was measured.

### Insertion capability of PTX/IR-780 SLNs @DMNs

To evaluate the *in vitro* skin insertion ability, the PTX/IR-780 SLNs @DMNs were pressed into the shaved dorsal rat skin or porcine skin for 5 min. The DMNs were removed, the insertion site stained with 1% trypan blue, and then imaged with a camera. To determine the insertion depth, the rat skin was prepared for histological specimens and observed under optical coherence tomography (OCT, HSO-2000, China). PTX/IR-780 SLNs @DMNs were inserted into the gelatin block with 35% water (w/w) to simulate DMN dissolution *in vitro*, and the morphology of DMNs in the gelatin block was observed with a microscope (Nikon, Tokyo, Japan) 50. DMNs were removed after 10 min and observed under a microscope along with the gelatin block. The gelatin block was observed again after 24 h to examine the accumulation of PTX/IR-780 SLNs at the application site.

### *In vivo* photothermal effect

To directly evaluate the photothermal effect of PTX/IR-780 SLNs @DMNs, a visual infrared thermal imaging camera (Tis75, Fluke, USA) was applied to detect the temperature changes in C57 mice bearing tumors under NIR laser irradiation. After removing the hair of mice at the dorsal flank, different DMNs were inserted into the skin by thumb pressure for 2 min. The mice were randomly divided into three groups: (1) control group, in which the mice were only exposed to NIR light; (2) blank DMN group, in which the mice were treated with DMNs without the drug and irradiated with the laser; and (3) PTX/IR-780 SLNs @DMN group, in which the mice were treated with PTX/IR-780 SLNs @DMNs and irradiated with laser for 5 min (808 nm, 1 W/cm^2^).

### *In vivo* imaging and biodistribution analysis

C57 female mice were injected with B16 cells (1 × 10^6^) in the dorsal flank to generate the subcutaneous melanoma mouse model. When the tumor reached 40 - 60 mm^3^, the mice were randomly divided into three groups: (1) treated with PTX/IR-780 SLNs @DMNs; (2) intratumorally injected with PTX/IR-780 SLNs; and (3) intravenously injected with PTX/IR-780 SLNs. Fluorescence signals at tumor sites were detected using a live fluorescence imaging system (IVIS Lumina XRMS, PerkinElmer, USA) at 0, 2, 4, 6, 8, 12, and 24 h. After 24 h, all mice were sacrificed, and the major organs (heart, liver, spleen, lung, and kidney) were collected to observe the fluorescence signal distribution.

### Expression of heat-shock protein 70 (Hsp70)

When the tumor reached 40 - 60 mm^3^, the C57 mice were randomly divided into three groups: (1) control group; (2) PTX/IR-780 SLNs @DMNs with laser irradiation for 5 min (808 nm, 1 W/cm^2^); and (3) PTX/IR-780 SLNs @DMNs without laser irradiation. Tumor tissues were collected 24 h after administration, fixed with 4% paraformaldehyde, and then evaluated for the expression of Hsp70 by immunofluorescence staining.

### *In vivo* anti-tumor study

A subcutaneous melanoma model was established by injecting B16 cells (1 × 10^6^) in the dorsal flank of C57 mice. When the tumors grew to 40 - 60 mm^3^, the mice were randomly divided into 8 groups: (1) no treatment; (2) PTX SLNs @DMNs; (3) IR-780 SLNs @DMNs + laser (++); (4) PTX/IR-780 solution @DMNs + laser (++); (5) PTX/IR-780 SLNs @DMNs + laser (+); (6) PTX/IR-780 SLNs intravenous injection + laser (++); (7) PTX/IR-780 SLNs intratumoral injection + laser (++); and (8) PTX/IR-780 SLNs @DMN + laser (++). “+” means laser irradiation for 5 min (1 W/cm^2^) on the day of administration, and “++” means laser irradiation for another 5 min (1 W/cm^2^) after 24 h of the first irradiation. The tumor volume and bodyweight of mice were monitored every other day, and the tumor volume was calculated by tumor length × tumor width^2^/2. The survival rate of the mice was recorded until death occurred or animals were euthanized when the tumor volume reached ≥ 2000 mm^3^.

On the 12th day after the treatments, mice were sacrificed, and the tumor and major organs were fixed in 4% paraformaldehyde, embedded in paraffin, and cut into 5 μm slices. The major organs were stained with hematoxylin-eosin (H&E) and observed under a microscope to assess formulations' safety. The tumors were stained with H&E, terminal deoxynucleotidyl transferase (TdT)-mediated dUTP nick end labeling (TUNEL), caspase 3, and Ki67 to further evaluate the therapeutic effects.

### Safety evaluation

To analyze skin recovery after application of DMNs, healthy C57 mice were divided into 3 groups: (1) no treatment; (2) PTX/IR-780 SLNs @DMNs without laser irradiation; and (3) PTX/IR-780 SLNs @DMNs with laser irradiation for 5 min (808 nm, 1 W/cm^2^). The skin recovery of mice was documented with a camera at 0, 1, 3, 7, and 10 days after administration.

To further evaluate the safety of various methods of administrations, mice were randomly divided into four groups for routine blood examination 51: (1) healthy mice; (2) intravenously injected with PTX/IR-780 SLNs; (3) intratumorally injected with PTX/IR-780 SLNs; and (4) PTX/IR-780 SLNs @DMNs. After treatment for 24 h, blood samples were collected from the eye socket vein and were used to evaluate the level of blood parameters.

### Statistical analysis

All data were presented as the mean ± the standard deviation (SD) of independent determinations. Statistical differences were considered significant when *P* < 0.05 and were calculated by using one-way analysis of variance (ANOVA) after the normality test and variance homogeneity test using SPSS (version 22.0, IBM Corporation, New York, USA).

## Results and Discussion

### Preparation and Characterization of PTX/IR-780 SLNs

**Figure 2:**
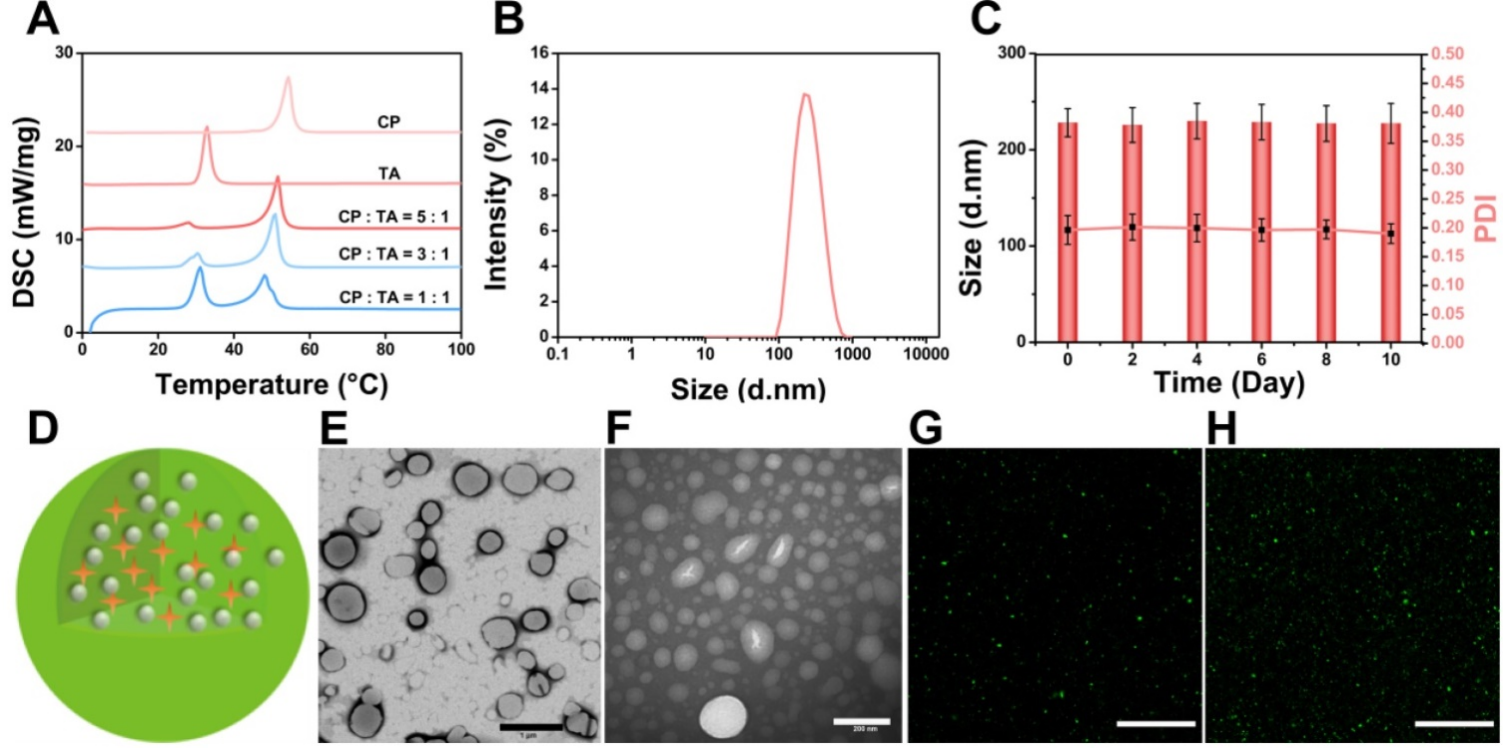
** Characterization of PTX/IR-780 SLNs.** (**A**) DSC thermograms of the CP, TA, and PTX/IR-780 SLNs with different ratios of CP to TA. (**B**) Size distribution of PTX/IR-780 SLNs. (**C**) Stability of the PTX/IR-780 SLNs at different time intervals (*n* = 3). (**D**) Structure illustration of PTX/IR-780 SLNs. (**E**) TEM image of PTX/IR-780 SLNs before irradiation; (scale bar: 1 µm). (**F**) TEM image of PTX/IR-780 SLNs after irradiation; (scale bar: 200 µm). (**G**) CLSM picture of PTX/IR-780 SLNs before irradiation; (scale bar: 1 µm). (**H**) CLSM picture of C6/IR-780 SLNs after irradiation; scale bar: 1 µm).

DSC was used to investigate thermal properties of the lipid materials to provide relevant physicochemical data of the melting point of the PTX/IR-780 SLNs. As shown in **Figure 2A**, the melting point of SLNs changed with different ratios of CP to TA, and the final formulation of the PTX/IR-780 SLNs was CP : TA = 5 : 1 with a melting point of 51.8 °C. DLS results showed that the average diameter of the PTX/IR-780 SLNs was approximately 230 nm **(Figure 2B)**. The particle size and polydispersity index of PTX/IR-780 SLNs remained constant when stored at 4 °C for 10 days **(Figure 2C)**, indicating good stability of the nanosystem. The schematic illustration of PTX/IR-780 SLNs is presented in **Figure 2D**. CP and TA served as the lipid materials of SLNs, and PTX and IR-780 were distributed in the matrix of SLNs. TEM images revealed that PTX/IR-780 SLNs were generally spherical **(Figure 2E)**. Upon irradiation with an 808 nm NIR laser (1 W/cm^2^) for 5 min, smaller particles were observed due to the phase transition effect **(Figure 2F)**. Moreover, the fluorescence signal of C6/IR-780 SLNs also became more dispersed after exposure to the NIR laser (1 W/cm^2^) for 5 min **(Figure 2G-H, Figure S1)** due to the release of C6 promoted by the laser-induced phase transition of the SLNs. The fluorescence spectra showed that free IR-780 solution presented a strong absorption peak at ~ 803 nm **(Figure S2A)** with a 10 nm redshift to 813 nm **(Figure S2B)**. Hydrophobic interactions and changes in solvent polarity might contribute to this phenomenon 24. The EE of the PTX and IR-780 measured by the ultrafiltration method was 97.41% ± 0.54 and 62.58% ± 0.98. The DL of the PTX and IR-780 was 2.97% ± 0.11 and 1.70% ± 0.11. It should be noted that the SLN preparation process involved relatively high temperature (70 °C), and thermal-sensitive drugs are unlikely compatible with this system. For those drugs, supercritical fluid technology and cold dispersion technology can be used to avoid the high temperature.

### Temperature-dependent and laser-triggered drug release

**Figure 3:**
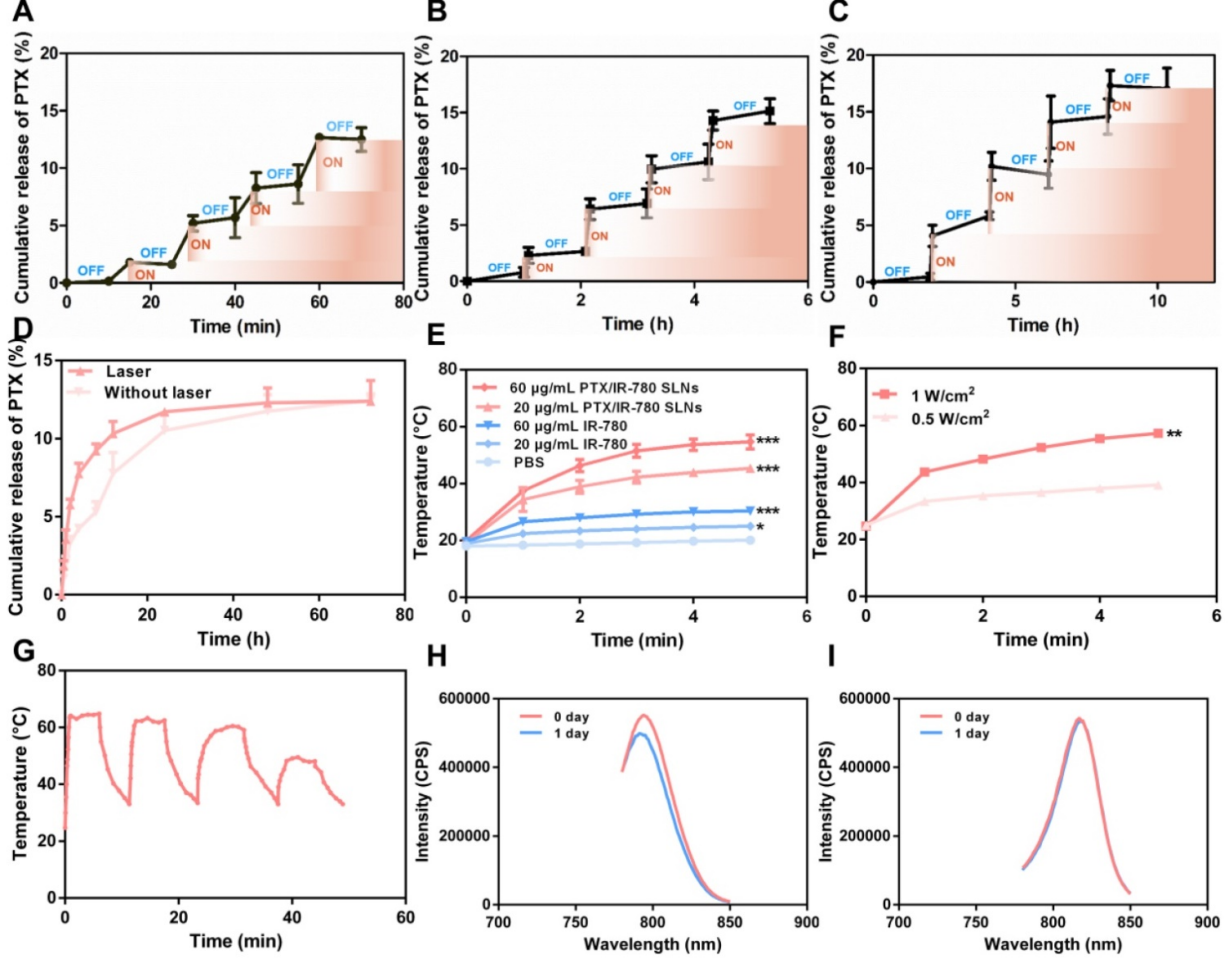
** Drug release profile and photothermal behavior of PTX/IR-780 SLNs.** PTX release profile from PTX/IR-780 SLNs triggered by temperature. The time interval between heating was (**A**) 10 min, (**B**) 1 h, and (**C**) 2 h. (**D**) PTX release profile from PTX/IR-780 SLN suspension after irradiation with 808 nm NIR at 1 W/cm^2^ for 5 min. (**E**) Photothermal effect of PTX/IR-780 SLNs and free IR-780 at different concentrations after irradiation with 808 nm NIR at 1 W/cm^2^ for 5 min. (**F**) Photothermal effect of PTX/IR-780 SLNs irradiated by 1 W/cm^2^ and 0.5 W/cm^2^ laser. (**G**) Thermal profile of PTX/IR-780 SLNs irradiated with 808 nm NIR at 1 W/cm^2^ for 5 min in 4 cycles. The fluorescence spectrum of (**H**) free IR-780 solution and (**I**) PTX/IR-780 SLNs after 1 day of storage in light. Data are expressed as the mean ± SD, *p < 0.05, **p < 0.01, ***p < 0.001 versus control (*n* = 3).

SLNs are usually prepared from lipid materials that melt at their melting points and re-solidify when the temperature drops. We speculated that the optimal SLNs would experience phase change at the high temperature (50 °C) induced by PTT and re-solidify when the temperature decreases at the end of laser irradiation. Therefore, temporally controlled temperature-dependent release profiles could be realized at different laser irradiation intervals. Here, we prepared SLNs using the CP and TA lipid materials whose melting points are 54 °C and 33 °C, respectively. Optimal SLNs with a melting point of 51.7 °C were obtained by systematically adjusting the ratio of CP to TA (5 : 1). **Figure 3A - C** displays the cumulative drug release profile of PTX when the time interval between heating was 10 min, 1 h, and 2 h. As expected, the pulsatile drug release profiles were achieved by the “ON” and “OFF” heating cycles. In each round of heating, the burst release amount of PTX was approximately 2% - 4%.

To evaluate the release profile after irradiation with a NIR laser, a specific amount of PTX/IR-780 SLNs was exposed to the 808 nm laser for a specific time. As shown in **Figure 3D**, a burst release was observed for the PTX/IR-780 SLNs with laser irradiation, followed by a slow release rate which was essentially the same as that for the PTX/IR-780 SLNs without laser exposure, and the cumulative release of PTX at 72 h was approximately 13%. Apparently, IR-780 absorbed the NIR laser energy and converted it into heat, inducing the phase transition of the SLNs and ultimately accelerating drug release from SLNs. When the laser was removed, the PTX/IR-780 SLNs suspension gradually cooled to room temperature, the SLNs re-solidified, and the drug release rate decreased. Therefore, different drug release profiles can be achieved by adjusting the laser “OFF” time and the number of irradiation cycles, realizing precise individualized medication.

### Photothermal properties of PTX/IR-780 SLN suspension

To evaluate the photothermal properties of PTX/IR-780 SLNs, both IR-780 solution and PTX/IR-780 SLNs were tested in PBS under 808 nm NIR light for 5 min. As shown in **Figure 3E**, the temperature of blank PBS had no change. Because of the low solubility and poor stability of IR-780 in water, the temperature of IR-780 solution slightly increased to 25.0 °C (IR-780: 20 μg/mL) and 30.4 °C (IR-780: 60 μg/mL). By contrast, the temperature of PTX/IR-780 SLNs suspension rapidly increased to 45.4 °C (IR-780: 20 μg/mL) and 54.7 °C (IR-780: 60 μg/mL) following irradiation. As displayed in **Figure 3F**, the maximum temperature of PTX/IR-780 SLNs increased from 35.1 °C to 54.7 °C when the laser power increased from 0.5 W/cm^2^ to 1 W/cm^2^. This indicated that the photothermal conversion efficiency was related to the concentration of IR-780 and the power intensity of NIR light. **Figure 3G** shows that after 3 cycles of irradiation, the temperature of PTX/IR-780 SLNs solution still rose to 50 °C, indicating that PTX/IR-780 SLNs could produce multiple photothermal effects. After 1 day of storage in light, the maximum absorption of IR-780 solution decreased **(Figure 3H)**. By contrast, the maximum absorption of PTX/IR-780 SLNs suspension showed no change **(Figure 3I)**. Following storage at 4 °C for 30 days, PTX/IR-80 SLNs showed no change while the IR-780 solution showed sedimentation **(Figure S3)**. These results indicated that encapsulation of IR-780 in SLNs improves its stability and maintains its photothermal property.

### *In vitro* cellular uptake and intracellular drug release

To determine whether B16 melanoma cells effectively uptake SLNs, C6 was labeled in SLNs to simulate chemotherapy drug PTX. After coculturing with C6/IR-780 SLN suspension for 2 h, sections of B16 cells were observed by CLSM, showing the uniform distribution of C6 and IR-780 in the cytoplasm of B16 cells **(Figure S4)**.

**Figure 4:**
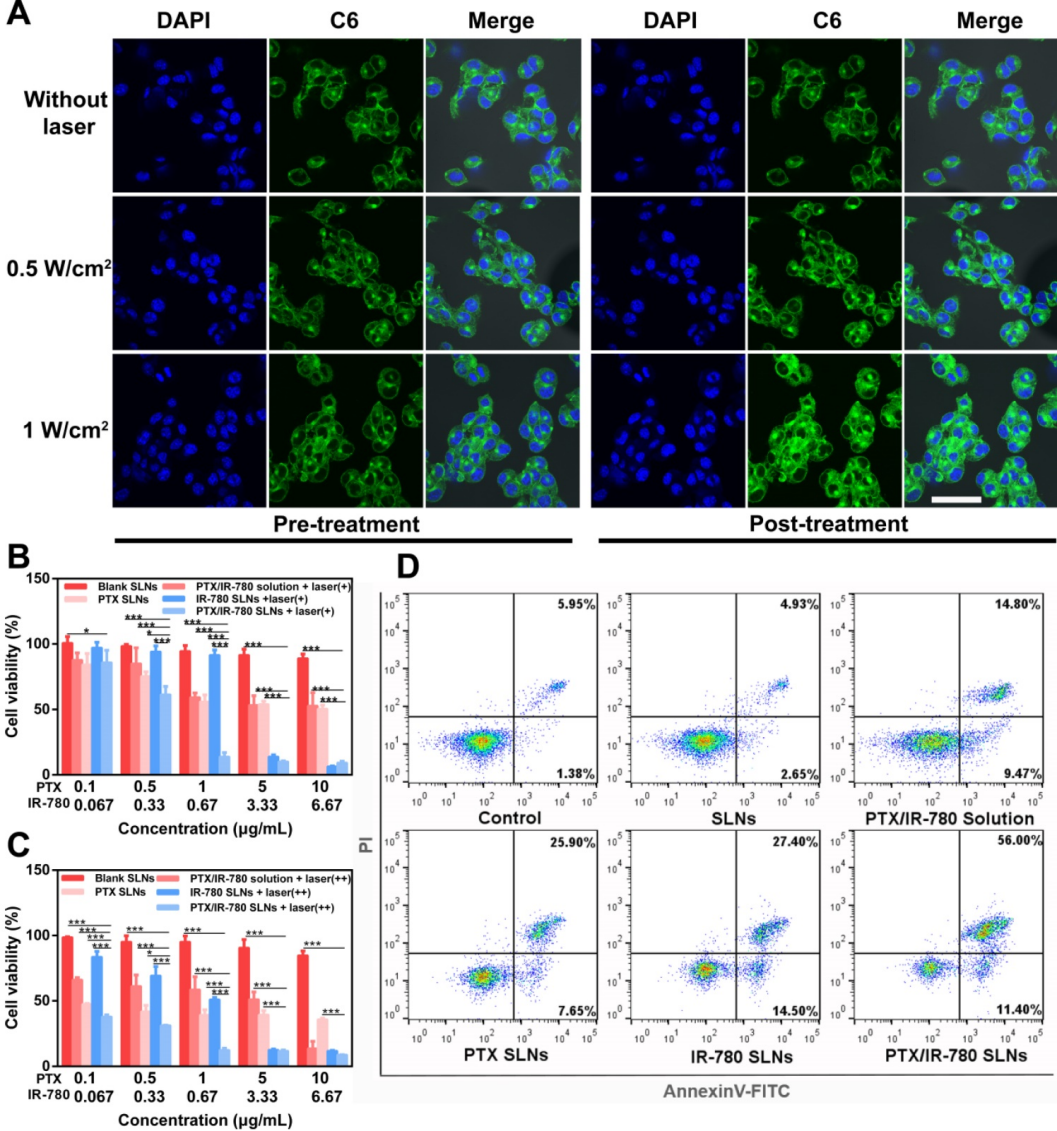
(**A**) Real-time images of intracellular drug release after laser irradiation for 5 min captured by CLSM (1 W/cm^2^ or 0.5 W/cm^2^). Cell nuclei were stained with DAPI (blue), and C6 is shown as green fluorescence (scale bar: 50 µm). Viability of cells treated with different formulations with or without laser irradiation for 5 min (800 nm, 1 W/cm^2^) and further incubated for (**B**) 24 h and (**C**) 48 h (*n* = 3). Data are expressed as the mean ± SD, *p < 0.05, **p < 0.01, ***p < 0.001 versus control (*n* = 3). The label “+” means laser irradiation for 5 min (1 W/cm^2^) on the day of administration, and “++” means laser irradiation for another 5 min (1 W/cm^2^) after 24 h of the first irradiation. (**D**) Apoptosis analysis by Annexin V-FITC/PI staining of B16 cells treated with PBS, SLNs, PTX SLNs, PTX/IR-780 solution + laser, IR-780 SLNs + laser, and PTX/IR-780 SLNs + laser (808 nm, 1 W/cm^2^).

The intracellular drug release triggered by NIR laser was then observed at the cellular level using CLSM. As shown in **Figure 4A,** C6 was released from SLNs when irradiated with 808 nm laser for 5 min. Compared with the untreated B16 cells, the laser group showed stronger C6 fluorescence signals. This was because the photothermal effect of IR-780 under NIR laser induced SLNs phase transition to release more C6. For the groups treated with 0.5 W/cm^2^ and 1 W/cm^2^ laser, the latter displayed a stronger C6 fluorescence signal than the former. As discussed above, 1 W/cm^2^ laser triggered higher temperature than 0.5 W/cm^2^, releasing more C6 from SLNs.

### *In vitro* cytotoxicity and apoptosis assay

To evaluate the cytotoxicity effect of PTX/IR-780 SLNs, B16 cells were treated with different formulations and the viability of cells was measured by CCK-8 at 24 h and 48 h. **Figure 4B - 4C** show that the cell viability in the presence of blank SLNs was higher than 85% after 24 h or 48 h of culture, indicating good compatibility of the SLNs. Since IR-780 is unstable in solution, the cell viability of the PTX/IR-780 solution group was almost the same as that for PTX SLNs after treatment for 24 h **(Figure 4B)**. For the PTX SLN group, the viability was 56% when the PTX concentration was 1 μg/mL. Further increase in PTX concentration had little effect with 50.5% viability observed at 10 μg/mL PTX concentration. Additionally, IR-780 alone had no cytotoxicity when the concentration was 0.67 μg/mL. By contrast, the viability of B16 cells sharply decreased to 14% in the PTX/IR-780 SLN group with 1 μg/mL PTX and 0.67 μg/mL IR-780. These results showed that chemo-photothermal therapy had better cell inhibition ability than chemotherapy or PTT alone. When the concentration of PTX and IR-780 increased to 10 and 6.67 μg/mL, the viability of cells treated with IR-780 SLNs was slightly lower than that of cells treated with PTX/IR-780 SLNs. This could be explained by the hyperpyrexia generated by the higher concentration of IR-780, almost entirely ablating the cancer cells. When the cells were irradiated again after 24 h and incubated for another 24 h, the cell viability significantly decreased **(Figure 4C)**, indicating that additional irradiation cycles further enhanced cytotoxicity. As shown in **Figure S5,** laser irradiation did not affect the cells as there was no difference in cell viability between the laser alone and control groups.

After treatment with different formulations, the cells were stained with Annexin V-FITC/PI. The results presented in **Figure 4D** were consistent with those of the CCK-8 assay, which further demonstrated that PTX/IR-780 SLNs exhibited superior anticancer effects due to the chemo-photothermal synergistic therapy.

### ROS generation in B16 cells

Several NIR dyes (such as IR-780 and IR-820) have excellent photophysical properties with significant shifts between absorption and emission bands and can potentially be used for photodynamic therapy 24, 52. When PTX/IR-780 SLNs were irradiated by 808 nm NIR laser, ROS was generated by IR-780 oxidized DCFH-DA into DCF and exhibited green fluorescence in B16 cells. As shown in **Figure S6**, there was no fluorescence in PBS or PTX/IR-780 solution with laser irradiation. Thus, the encapsulated IR-780 in SLNs could generate ROS and exhibit photodynamic properties.

### Formulation and characterization of PTX/IR-780 SLNs @DMNs

**Figure 5:**
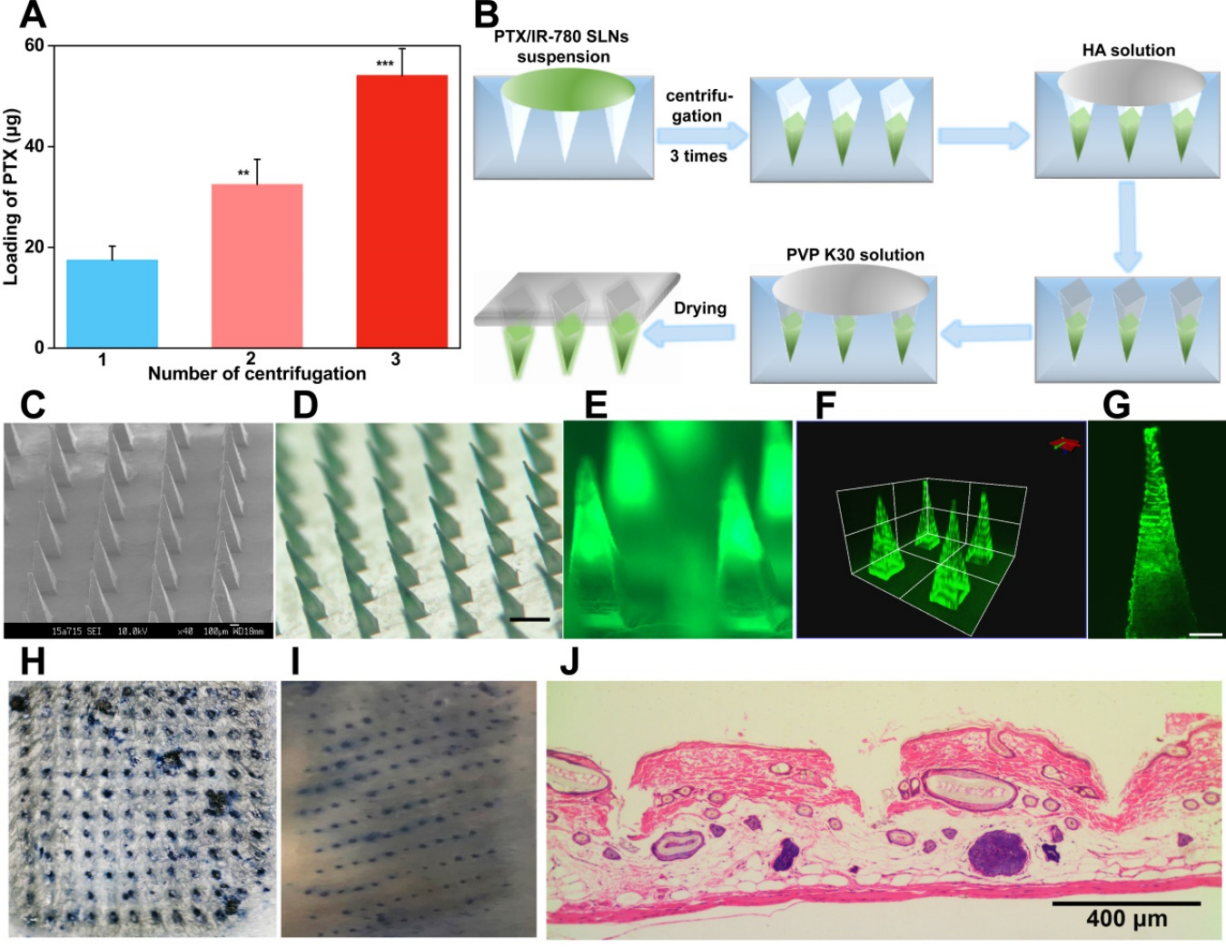
** Characterization of PTX/IR-780 SLNs @DMNs.** (**A**) Drug-loading amount of PTX in DMNs prepared with different centrifugation times. Data are expressed as the mean ± SD, *p < 0.05, **p < 0.01, ***p < 0.001 versus control (*n* = 3). (**B**) Preparation scheme of PTX/IR-780 SLNs @DMNs. (**C**) SEM image of PTX/IR-780 SLNs @DMNs. (**D**) Photograph of PTX/IR-780 SLNs @DMNs under bright field (scale bar: 500 µm), and (**E**) fluorescence microscopy (**F**) 3D reconstruction image of PTX/IR-780 SLNs @DMNs captured by CLSM and (**G**) single needle image (scale bar: 100 µm). (**H**) Dorsal rat skin of a shaved area after insertion of PTX/IR-780 SLNs @DMNs. (**I**) Porcine skin after insertion of PTX/IR-780 SLNs @DMNs. (**J**) Corresponding histological section of inserted rat skin.

Insufficient drug loading of DMNs is still one of the greatest challenges in application due to the relatively low needle volume. In this study, a multiple centrifugation molding technology was employed to concentrate drug-loaded nanoparticles into the needle tips as described in our previous study 49. **Figure 5A** shows that the loading amount of PTX in DMNs increased with an increase in centrifugation times. In particular, the PTX loading amount reached 54.13 μg per patch after three centrifugation cycles, which was 1.7- and 3.1-fold higher than that of two (32.50 μg per patch) and one centrifugation (17.46 μg per patch) cycles. Considering drug loading efficiency and the convenience of the preparation, we selected 3 centrifugation cycles to enrich SLNs at the tip of the microneedles **(Figure 5B)**. The encapsulation and loading efficiency of SLNs in the DMNs were 84.12% ± 8.21 and 12.32% ± 1.29, respectively. The SEM image of PTX/IR-780 SLNs @DMNs **(Figure 5C)** depicted that the quadrangular pyramid-shaped needles were uniformly distributed on the base substrate with a height of 800 μm and tip-to-tip space of 750 μm. The stereo fluorescence photographs of PTX/IR-780 SLNs @DMNs under bright field and fluorescence microscopy are shown in **Figure 5D** and **Figure 5E**. The 3D simulation (**Figure 5F**) and the fluorescence image of PTX/IR-780 SLNs @DMNs captured by CLSM (**Figure 5G**) are also presented. These results revealed that the PTX/IR-780 SLNs were mainly concentrated in the tips of needles, which was desirable to efficiently deliver the drug-loaded nanoparticles into the lesion site. The particle size of SLNs released from DMNs was measured as 250 nm (**Figure S7**), indicating that the physical characteristics of SLNs were maintained after packaging into DMNs.

### Insertion capability of PTX/IR-780 SLNs @DMNs

Skin penetration of microneedles is a prerequisite for effective drug delivery. HA is an endogenous component of the human body 53. DMNs fabricated with HA have been demonstrated to exhibit enough mechanical strength to pierce into the skin and subsequently dissolve quickly by interstitial fluid 4. In this study, the PTX/IR-780 SLNs @DMNs using HA as the polymer matrix were pressed into the dorsal skin for 2 min. After the removal of DMNs, the inserted site was stained with a dye. As shown in **Figure 5H & 5I**, a complete array of spots corresponding to the DMN puncture sites were clearly observed, indicating that the DMNs were successfully inserted into the skin. The H&E-stained section demonstrated that PTX/IR-780 SLNs @DMNs were completely inserted and embedded within the tissue **(Figure 5J)**. To further determine the insertion depth, the OCT was used to obtain the real-time images of the insertion process in the dorsal rat skin. As shown in **Figure S8**, optical signals were detected at a depth of approximately 700 μm. It is of note that the insertion depth in H&E-stained sections was only 200 - 220 μm due to skin elastic deformation during skin section preparation. **Figure S9** shows that the PTX/IR-780 SLNs @DMNs completely dissolved after insertion into gelatin blocks **(Figure S9B)**. Following removal of the base of DMNs, PTX/IR-780 SLNs remained in the pore channel of gelatin blocks created by DMNs even after 24 h **(Figure S9F)**. Collectively, these results indicated that PTX/IR-780 SLNs @DMNs could quickly release SLNs at the tumor site to achieve long-term treatment.

### *In vivo* photothermal effect at the tumor site

**Figure 6:**
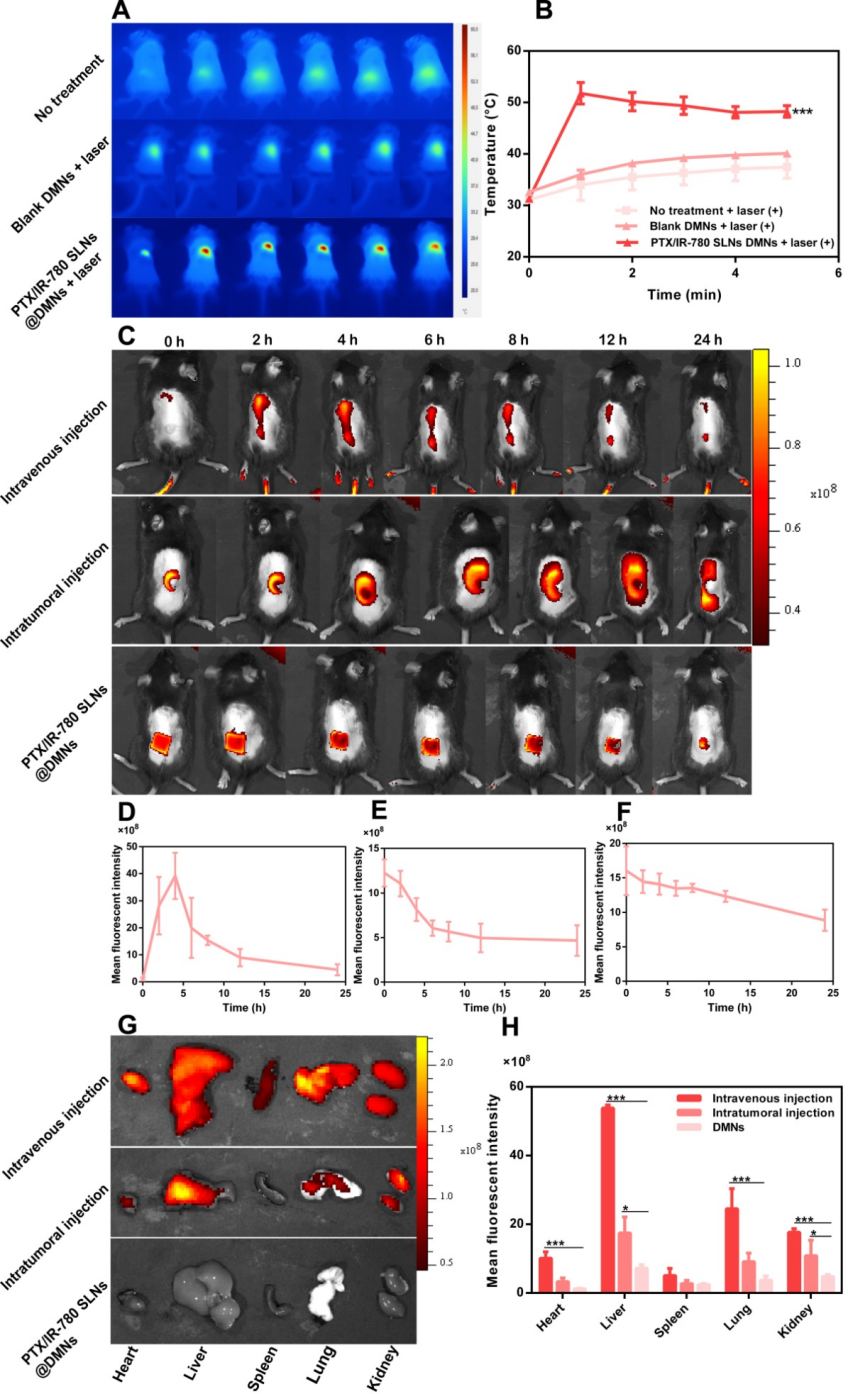
***In vivo* photothermal effect and biodistribution analysis.** (**A**) Infrared thermal images of PTX/IR-780 SLNs @DMNs in treated mice. (**B**) Temperature change curve at the irradiation site (808 nm, 1 W/cm^2^) (*n* = 3). (**C**) Fluorescent image of B16 tumor-bearing mice after administration of PTX/IR-780 SLNs by intravenous injection, intratumoral injection, and DMNs over 24 h. (**D - F**) Mean fluorescent intensity at different time intervals after administration of PTX/IR-780 SLNs by intravenous injection, intratumoral injection, and DMN (*n* = 3). (**G**) Fluorescent image and (**H**) mean fluorescent intensity of major organs 24 h post administration (*n* = 3). Data are expressed as the mean ± SD, *p < 0.05, **p < 0.01, ***p < 0.001 versus control.

The *in vivo* photothermal effect of PTX/IR-780 SLNs @DMNs was evaluated using an infrared thermal imaging camera to record the local temperature every minute **(Figure 6A - B)**. The initial temperature for the control and blank DMN groups was 31.3 °C and 32.6 °C, respectively. Upon irradiation with 808 nm NIR laser, the local temperature of the tumor only slightly increased (37.4 °C and 40.1 °C, respectively), and the temperatures were lower than the critical temperature value for irreversible cellular damage (e.g., hyperthermia temperature at 42 °C) 4. By contrast, the local temperature increased from 31.3 °C to 51.8 °C after treatment with PTX/IR-780 SLNs @DMNs plus irradiation. These results indicated that NIR alone or blank DMNs did not reach the critical value of photothermal therapy, while the local temperature at tumor site reached an ideal photothermal effect when PTX/IR-780 SLNs @DMNs were applied. The expression level of Hsp70 was related to the increased temperature at the local site 54. In our experiment, a strong positive expression of Hsp70 was observed after treatment with PTX/IR-780 SLNs @DMNs with laser irradiation for 5 min **(Figure S10)**, indicating that irradiation with 808 nm laser induced IR-780 and increased the tumor site temperature.

### *In vivo* imaging and biodistribution analysis

To investigate the duration of PTX/IR-780 SLNs' accumulation at the tumor site, the fluorescence signal variation in B16 melanoma-bearing mice at different time points was examined through IVIS **(Figure 6C - 6F, S11)**. A strong fluorescence signal was observed at the tumor site 4 h after intravenous injection of PTX/IR-780 SLNs and then the fluorescence signal gradually decreased. Therefore, the laser was applied 4 h after intravenous injection in the anti-tumor study. For the DMN and intratumoral injection groups, the strongest fluorescence signal was observed at the tumor site at 0 h. After 12 h, the relative fluorescence signal accumulated at the tumor site in the intratumoral injection group was 40.52%. By contrast, the relative fluorescence signal accumulated at the tumor site of the DMN group was 76.87%. To further study the biodistribution of PTX/IR-780 SLNs after various administrations, major organs (heart, liver, spleen, lung, and kidney) were collected 24 h post-administration **(Figure 6G - H)**. The DMN group did not show any fluorescence signal in the organs, while an extensive fluorescence signal was found in the intratumoral and intravenous injection groups. These results showed that compared with intratumoral and intravenous injection, DMNs provided a stable “Zone accumulation” to constrain the drug at the tumor site for a longer period of time. This was because PTX/IR-780 SLNs @DMNs could create hundreds of microchannels at the tumor site, uniformly release PTX/IR-780 SLNs and form multiple *in-situ* drug reservoirs. This regional delivery strategy is desirable for effective therapy of superficial tumors.

### *In vivo* anti-melanoma tumor study

**Figure 7:**
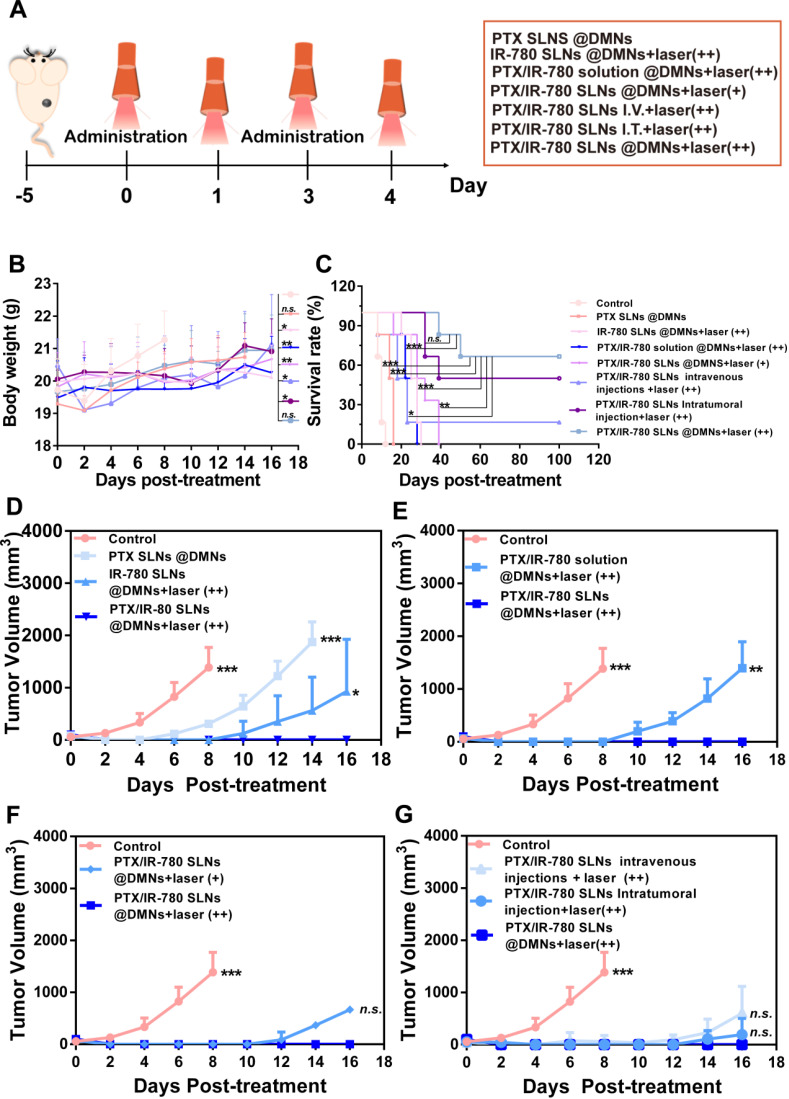
***In vivo* anticancer effect of various treatments in subcutaneous B16 tumor-bearing C57 mice.** (**A**) Schematic diagram of the therapeutic model of B16 tumor. I.V. means intravenous injection, I.T. means intratumoral injection. (**B**) Bodyweight and (**C**) Survival rate of mice after various treatments. Tumor volume after various treatments for 14 days: (**D**) Groups treated with PTX SLNs @DMNs, IR-780 SLNs @DMNs + laser (++), and PTX/IR-780 SLNs @DMNs + laser (++); (**E**) Groups treated with PTX/IR-780 solution @DMNs + laser (++) and PTX/IR-780 SLNs @DMNs + laser (++); (**F**) Groups treated with PTX/IR-780 SLNs @DMNs + laser (+) and PTX/IR-780 SLNs @DMNs + laser (++); (**G**) Groups treated with DMNs, intratumoral injection, and intravenous injection. Values are expressed as the mean ± S.D., *n.s.*, not significant, *p < 0.05, **p < 0.01, ***p < 0.001 versus group of PTX/IR-780 SLNs @DMNs (++).

Encouraged by the satisfactory *in vitro* therapeutic effect and the favorable NIR-induced hyperthermia *in vivo*, we evaluated the anticancer effect of the PTX/IR-780 SLNs @DMNs in B16 tumor-bearing mice. Tumor-bearing C57 mice were randomly divided into 8 groups. The tumor volume, survival rate, and bodyweight of treated mice were recorded every other day. **Figure 7A** displays the schematic diagram for the therapy of the B16 tumor model. As is evident from **Figure 7B**, the weight of mice did not significantly change during the entire test period except for mice in the control group, probably due to the growing tumor burden. This observation provided evidence for good biocompatibility and safety of the designed drug delivery system. Major organs of the mice were also collected on day 12 and stained with H&E. No noticeable pathological abnormalities in the heart, liver, spleen, lung, and kidney were observed after various treatments **(Figure S12)**, further demonstrating low systemic toxicity of PTX/IR-780 SLNs and SLNs loaded DMNs.

As shown in **Figure 7D**, the tumor volume in mice without any treatment increased sharply and reached approximately 1500 mm^3^ on day 8. The tumors in mice treated with PTX SLNs @DMNs recurred on day 4, with the tumor volume reaching 1850 mm^3^ on day 14. For the IR-780 SLNs @DMNs + laser (++) group, tumor recurrence occurred on day 8, and the tumor volume was 930.38 mm^3^ on day 16. This indicated that the anti-tumor effect of PTT was better than that of chemotherapy. For the PTX/IR-780 SLNs @DMNs + laser (++) group, the primary tumor was completely eradicated with a curable rate of 100% **(Figure 7C)** on day 16. There was no tumor recurrence through day 30, which confirmed the highly significant anti-tumor effect of the nanosystem. Due to the instability of IR-780 in aqueous solution, the tumor volume in mice treated with PTX/IR-780 solution DMNs + laser (++) was 1394.75 mm^3^ on day 16 **(Figure 7E)**, and the anti-tumor effect was far worse than that of PTX/IR-780 SLNs @DMNs +laser (++).

In this study, PTX/IR-780 SLNs @DMNs (+) and PTX/IR-780 SLNs @DMNs (++) were used in tumor-bearing mice to evaluate the multiple light treatment effect. As shown in **Figure 7F**, following one-time laser irradiation, the tumor recurred on day 12. Strikingly, when the second irradiation was added 24 h after the first irradiation, no tumor recurrence occurred through 30 days, which could be explained by the following reasons. First, the heat generated by NIR directly kills cancer cells through thermal ablation.

Second, the PTX release rate is accelerated by NIR irradiation. Moreover, increasing local temperature also disrupts cellular membranes to increase the cellular uptake of SLNs. Therefore, the improved efficacy was realized with a lower dose due to synergistic effects compared to monotherapy of PTX or NIR. Dong et al. also developed microneedles to deliver the anticancer drug doxorubicin and gold nanocage for melanoma treatment with four administrations 4. In our study, however, only two administrations were required to effectively eliminate the tumor and inhibit recurrence. Thus, PTX/IR-780 SLNs @DMNs provide an ideal topical delivery strategy to attain temporally controlled repeated dosing with a single administration.

First proposed in the 1970s, microneedles represent a new generation of transdermal delivery approach to many fields. In this study, DMNs were employed to directly deliver nanoparticles into a superficial tumor site with three-dimensional drug distribution. We used both intravenous and intratumoral injections to systematically compare their antitumor effects and the results are shown in **Figure 7G**. Compared with intravenous and intratumoral injections, the mice treated with PTX/IR-780 SLNs @DMNs + laser (++) prevented tumor recurrence and had the highest survival rate of 66.67% after 100 days of treatment **(Figure 7C)**. The reason that some mice did not survive in the PTX/IR-780 SLNs @DMNs + laser (++) group could be due to the individual differences of the mice, or the mice died due to other diseases. It should be noted that the drug dose delivered with intravenous injection was 5-fold higher than that of DMNs. These results were consistent with the *in vivo* imaging and biodistribution results. “Zone accumulation” of DMNs increased drug utilization in the lesion because of spatially controlled uniform distribution, ultimately resulting in superior antitumor efficacy. This is a promising approach for reducing the dose and administration frequency of chemotherapeutic drugs in clinical treatment.

**Figure 8:**
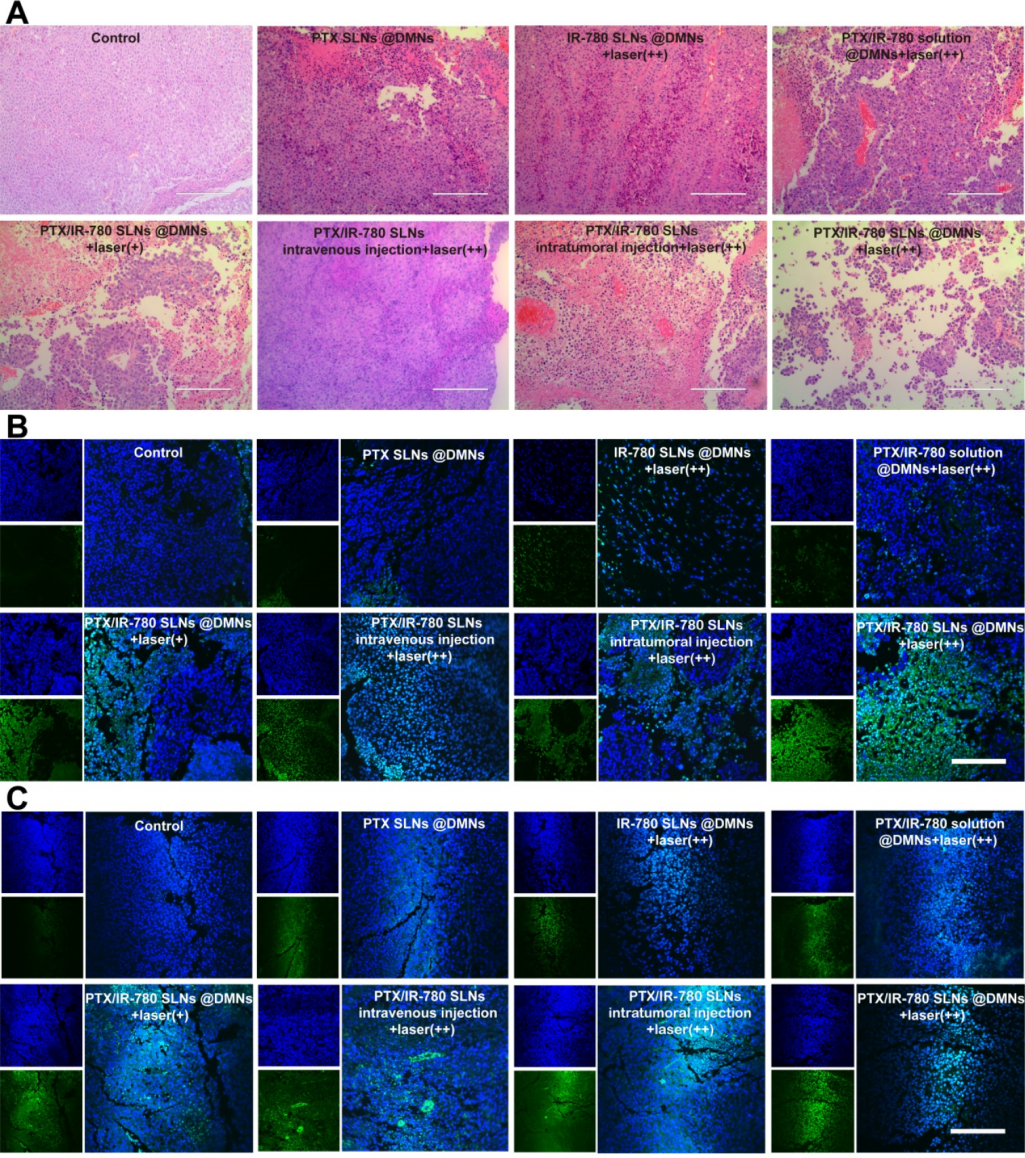
(**A**) H&E staining, (**B**) TUNEL immunofluorescence staining, and (**C**) Caspase 3 immunofluorescence staining of tumor tissues after various treatments. (scale bars: 200 µm).

We performed H&E staining of the tumor sections to further evaluate the anticancer effect of various treatments. As shown in **Figure 8A**, tumor cells were abundantly in present in the control group, while cell shrinkage and absence of nuclei were observed in the treated groups, indicating severe cell damage. Especially in the PTX/IR-780 SLNs @DMNs + laser (++) group, most B16 cells were damaged. The induced apoptosis of tumor cells was evaluated by TUNEL **(Figure 8B)**; compared with the other treated groups, tumor-bearing mice showed the highest number of apoptotic cells (green fluorescence) after treatment with PTX/IR-780 SLNs @DMNs + laser (++). Since the activation of caspase 3 is associated with apoptosis in cells, we examined the expression of caspase 3 in tumor tissues after tumor-bearing mice treating for 12 days. Immunofluorescence staining showed that caspase 3 was markedly upregulated after PTX/IR-780 SLNs @DMNs + laser (++) treatment **(Figure 8C)**. Ki67 expression is a marker of tumor cell proliferation 55. **Figure S13** shows decreased Ki67 in the PTX/IR-780 SLNs @DMNs + laser (++) group. **Figure S14** shows a lack of expression of Ki67 in healthy mice skin**,** indicating the effect of the treatment on inhibiting tumor proliferation. Collectively, the results confirmed that PTX/IR-780 SLNs @DMNs + laser (++) has a superior effect in suppressing tumors.

### Safety evaluation

**Figure S15** displays the skin recovery of mice after treatment with PTX/IR-780 SLNs @DMNs. For the group without irradiation, the treated site showed clear puncture spots caused by DMN insertion that recovered within 1 - 3 days. For the group with irradiation, the local site was slightly red at the beginning because of the relatively high temperature. The red color was replaced by a scab on day 3, which gradually fell off within 7 - 10 days, without leaving a noticeable scar. These results indicated that PTX/IR-780 SLNs @DMNs with irradiation are safe and tolerable.

**Figure S16** and **Figure S17** show blood safety after various treatments. The red blood cells, platelets, and hemoglobin levels did not show obvious changes in any of the groups. However, the intravenous injection group had a high level of lymphocytes and low level of neutrophils compared with other groups. This is because PTX causes strong myelosuppression, and systemic circulation and intravenous injection aggravated this side effect. In contrast to intravenous injection, PTX/IR-780 SLNs accumulated at the local site to avoid myelosuppression in the DMNs and intratumoral injection groups. The routine blood examination results demonstrated that PTX/IR-780 SLNs @DMNs is a safe treatment for tumor therapy.

## Conclusion

In this study, dissolving microneedles were used to directly deliver laser-inducible transformable SLNs into a superficial tumor site with spatiotemporally controlled pulsatile release properties. PTX/IR-780 SLNs @DMNs + laser (++) significantly inhibited tumor growth with a curable rate of 100% in the B16 cell tumor-bearing model. The SLNs were conducive to maintaining IR-780 stability and providing a platform for hydrophobic drug loading into DMNs. Furthermore, controlled burst release and multiple PTT achieved the best therapeutic outcome. Finally, PTX/IR-780 SLNs @DMNs combined the advantages of SLNs and DMNs, exerting a synergistic chemo-photothermal effect on tumor inhibition. Future studies, including experiments on primates and even human volunteers, are necessary to advance the clinical application of microneedles.

## Supplementary Material

Supplementary figures.

## Abbreviations

PTX: paclitaxel
DMNs: dissolving microneedles
SLNs: solid lipid nanoparticles
PTT: photothermal therapy
NIR: near-infrared
TA: tricaprin
CP: Cetyl Palmitate
HA: Sodium hyaluronic acid
PVP K90: polyvinyl pyrrolidone
PDMS: polydimethylsiloxane
FBS: fetal bovine serum
CCK-8: counting Kit-8
Annexin V-FITC/PI: Annexin V-fluorescein isothiocyanate (FITC)/PI
DSC: differential scanning calorimetry
DLS: dynamic light scattering
TEM: transmission electron microscope
C6: coumarin-6
CLSM: confocal laser scanning microscopy
HPLC: high-performance liquid chromatography
EE: encapsulation efficiency
PBS: phosphate-buffered saline
DAPI: 4', 6-diamidino-2-phenylindole
SEM: scanning electron microscope
OCT: optical coherence tomography
IVIS: live fluorescence imaging system
Hsp70: heat-shock protein 70
