# Supplementary material for: Dissolving Microneedles with Spatiotemporally controlled pulsatile release Nanosystem for Synergistic Chemo-photothermal Therapy of Melanoma
Source: Theranostics. 2020 Jul 9;10(18):8179–96. doi: 10.7150/thno.44194 (PMC7381723; doi:10.7150/thno.44194)
Supplement: Supplementary file 1 — Supplementary figures. [file thnov10p8179s1.pdf]

Dissolving microneedles with spatiotemporally controlled pulsatile release nanosystem for synergistic chemo-photothermal therapy of melanoma

Wanbing Qin[1#], Guilan Quan[2#], Ying Sun[1], Minglong Chen[1], Peipei Yang[3], Disang Feng[2], Ting Wen[1], Xinyu Hu[1], Xin Pan[1✉], Chuanbin Wu[1, 2]

1. School of Pharmaceutical Sciences, Sun Yat-sen University, Guangzhou 510006, China

2. College of Pharmacy, Jinan University, Guangzhou, 510632, China

3. Department of PharmacyGuangzhou Women and Children's Medical Center, Guangzhou Medical University, Guangzhou 510623, China

[#] These authors contributed equally to this work.

✉ Corresponding author: Xin Pan (panxin2@mail.sysu.edu.cn)

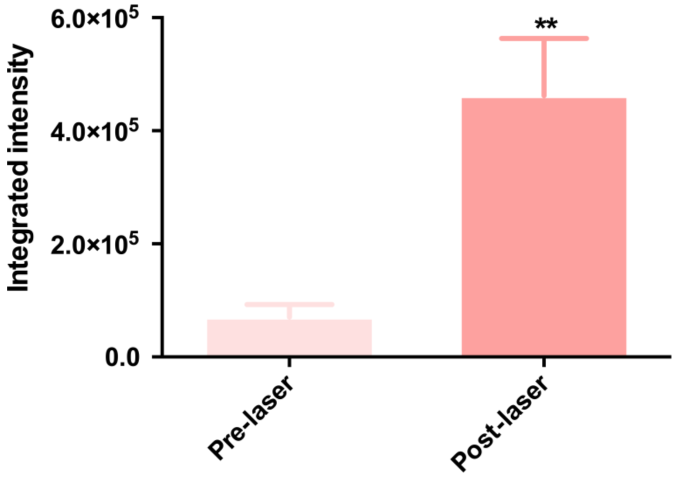

Figure S1. Quantitative analysis of fluorescence intensity of C6 before and after laser treatments.

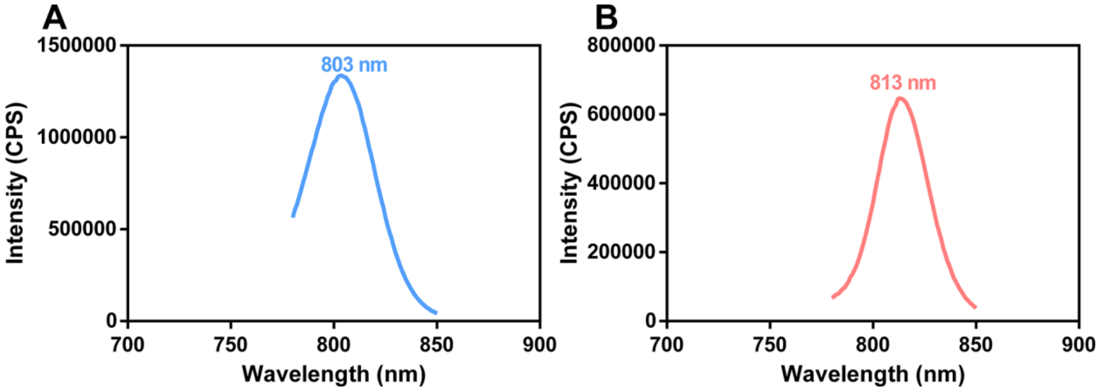

Figure S2. Fluorescence spectra of (A) IR-780 solution and (B) IR-780 SLNs suspension.

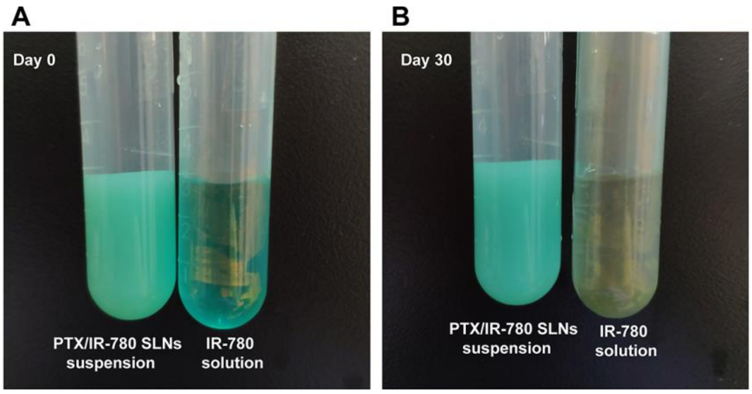

Figure S3. The picture of PTX/IR-780 SLNs suspension and IR-780 solution placed at 4 °C for (A) 0 day and (B) 30 days, respectively.

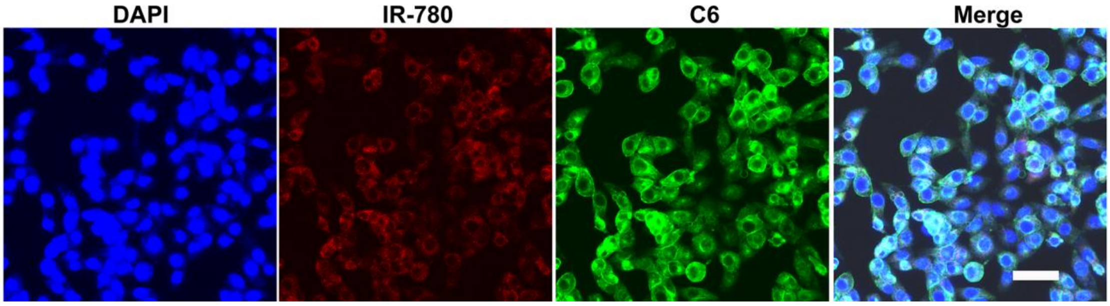

Figure S4. The CLSM images of B16 cells incubated with C6/IR-780 SLNs for 2 h (IR-780: 10 μg/mL, C6:1 μg/mL). The scale bar is 50 μm.

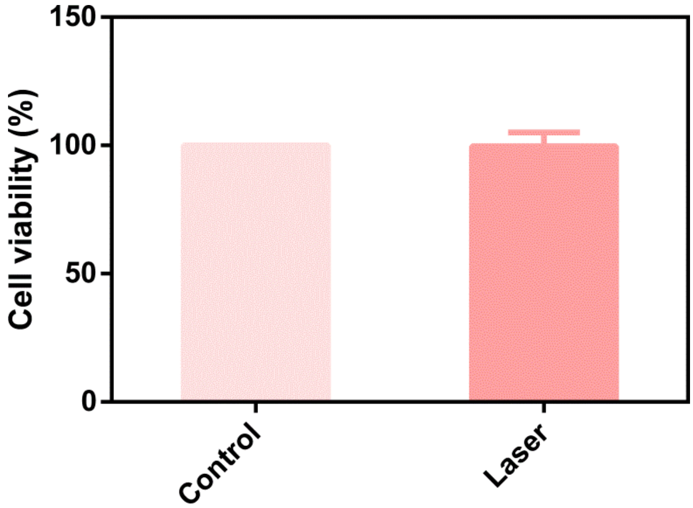

Figure S5. Viability of B16 cells treated with or without laser irradiation (n = 3).

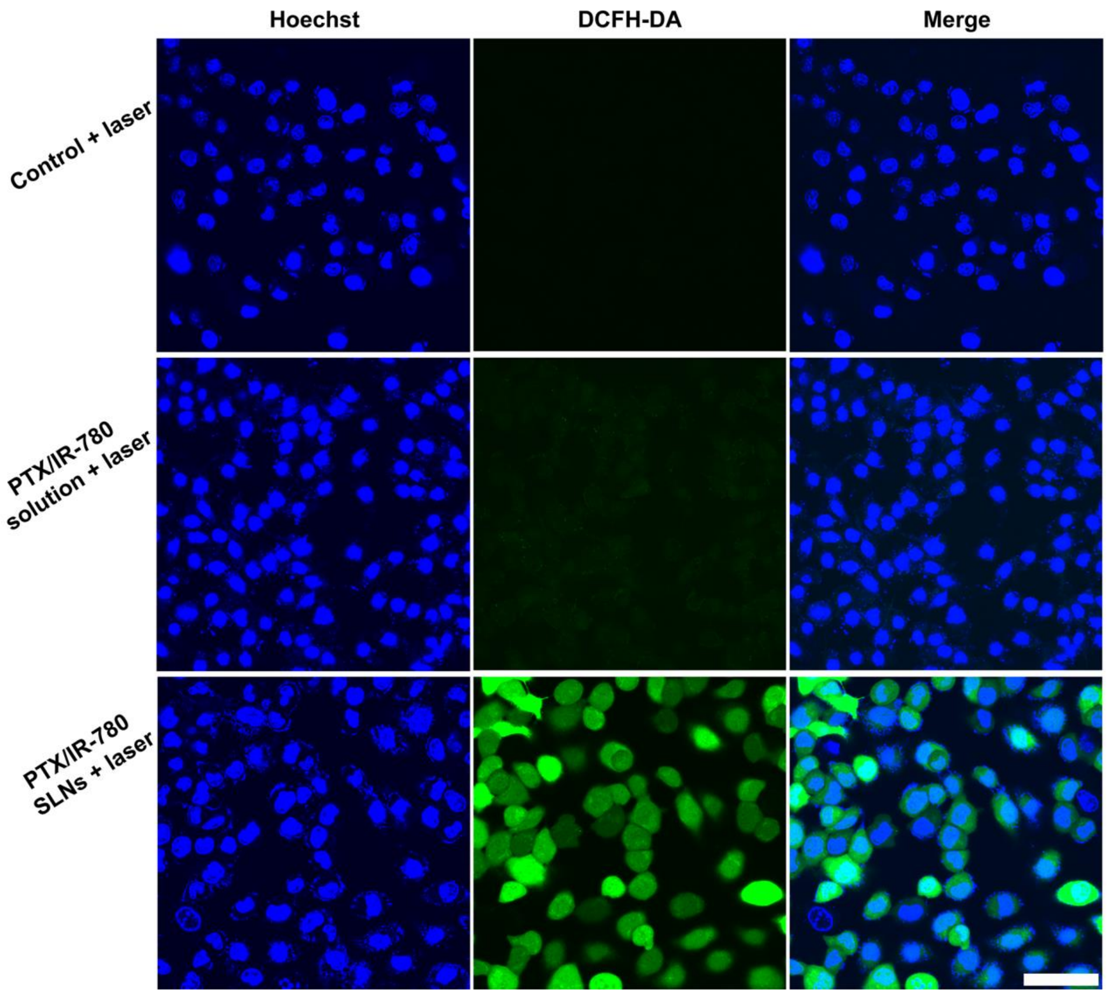

Figure S6. The CLSM images of ROS generation in B16 cells after treated with different treatments with laser (808 nm, 1W/cm^2). The scale bar is 50 μm.

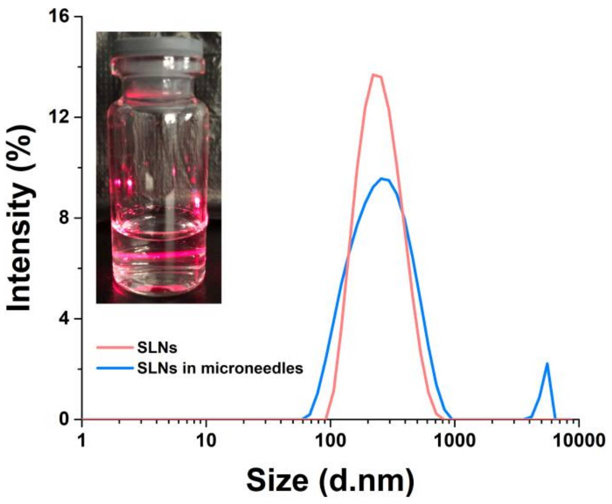

Figure S7. The particle size of SLNs before and after dissolving PTX/IR-780 SLNs @DMNs

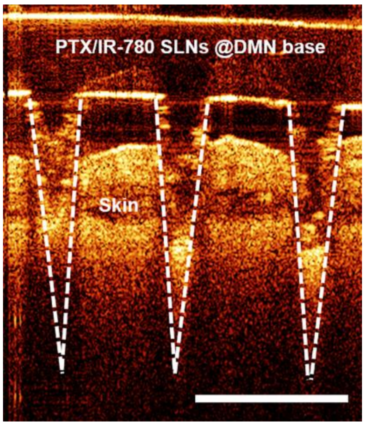

Figure S8. Two-dimentional OCT image of rat skin after inserted with PTX/IR-780 SLNs @DMNs. The scale bar is 500 μm.

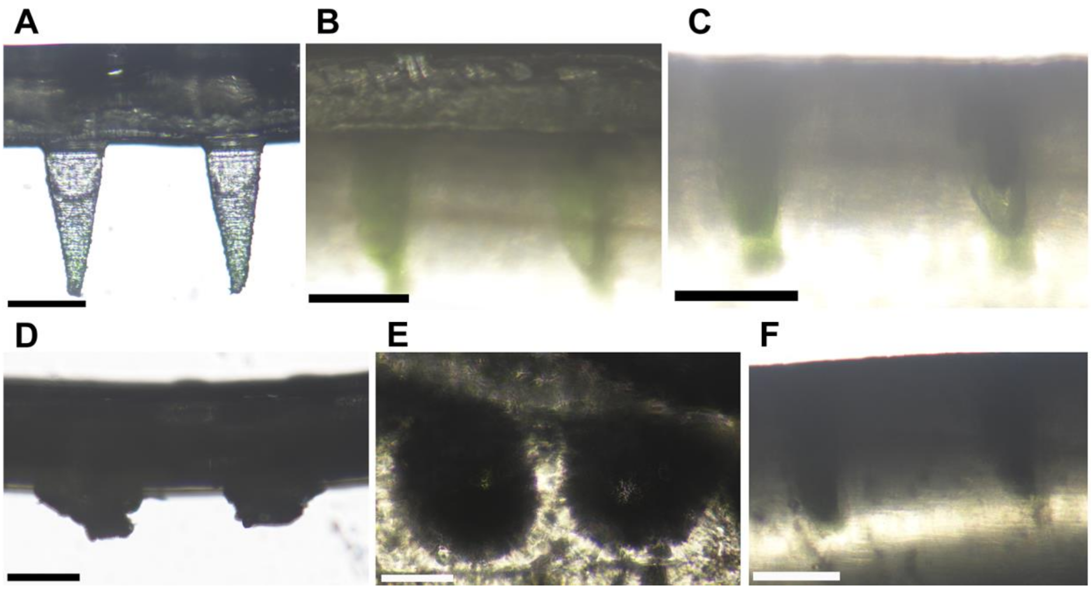

Figure S9. Bright field micrographs of PTX/IR-780 SLNs @DMNs inserted into the gelatin gel. (A) The picture of PTX/IR-780 SLNs @DMNs before insertion. (B) The picture of PTX/IR-780 SLNs @DMNs inserted into the gelatin block. (C) The picture

of PTX/IR-780 SLNs accumulated in the gelatin block after the DMNs base part was removed. (D) The picture of PTX/IR-780 SLNs @DMNs after removed from gelatin block. (E) The surface of the gelatin block after removed PTX/IR-780 SLNs @DMNs. (F) PTX/IR-780 SLNs accumulated in the gelatin block after sealed for 24 h. The scale bar is 400 μm.

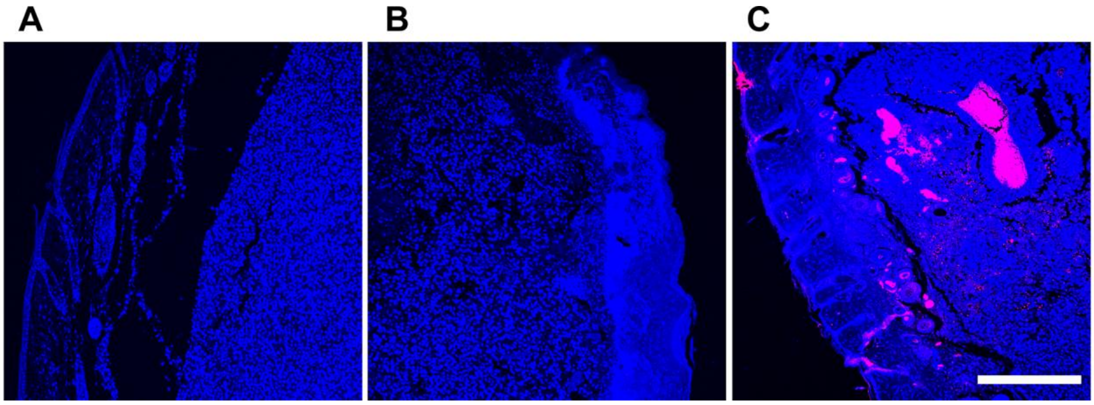

Figure S10. HSP 70 immunofluorescence staining of tumor after different treatments with or without laser (808 nm, 1 W/cm^2). (A) No treatment. (B) PTX/IR-780 SLNs @DMNs without laser. (C) PTX/IR-780 SLNs @DMNs laser (++). The scale bar is 400 μm.

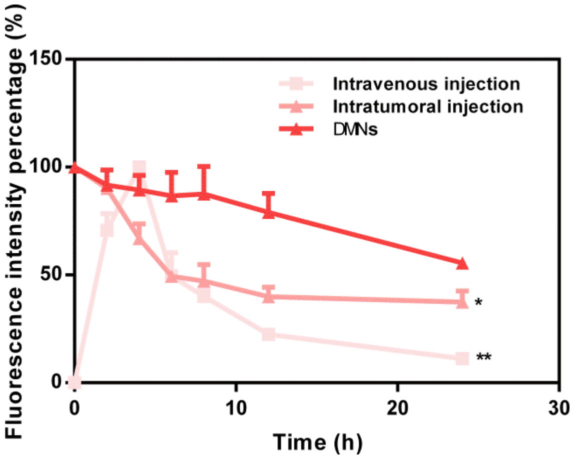

Figure S11. Normalized value of mean fluorescent intensity at different time intervals

after administration of PTX/IR-780 SLNs by intravenous injection, intratumoral injection, and DMN, respectively (n = 3). Data are expressed as the mean ± SD, *p < 0.05, **p < 0.01, ***p < 0.001 versus DMNs.

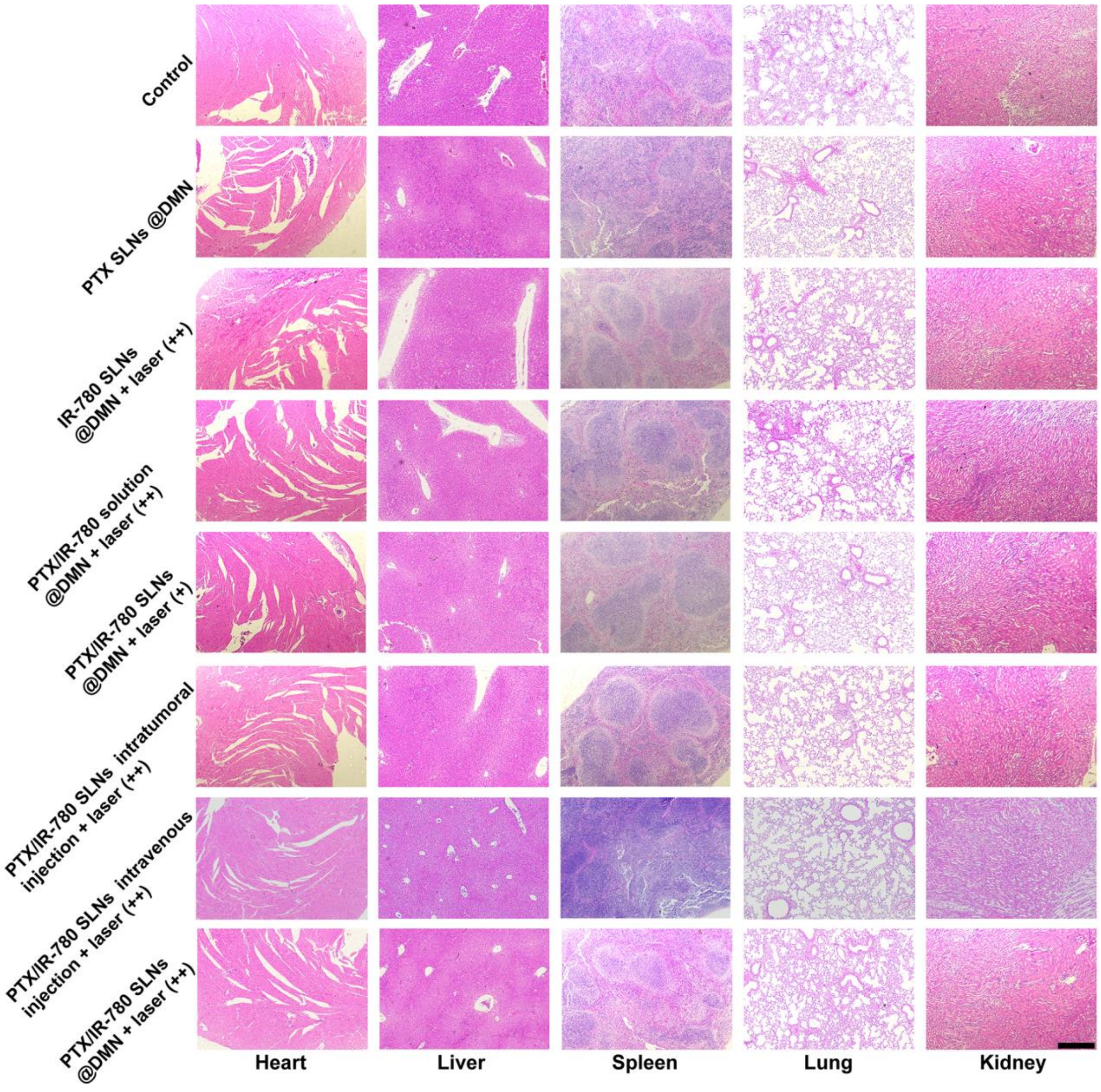

Figure S12. H&E staining images of major organs after different treatments. The scale bar is 100 μm.

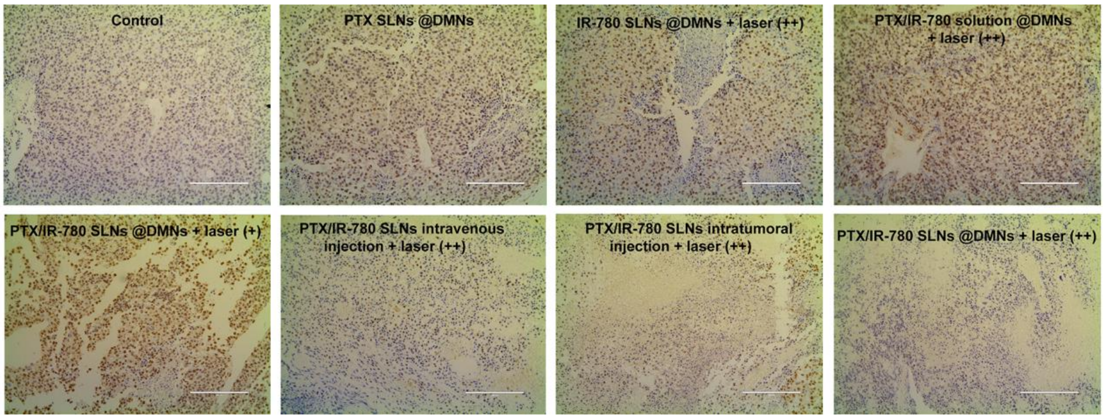

Figure S13. Ki67 staining of tumors after different treatments for 12 days. The scale bar is 400 μm.

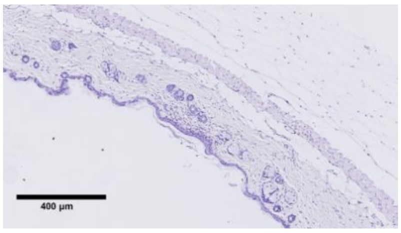

Figure S14. Ki67 staining of healthy mice skin.

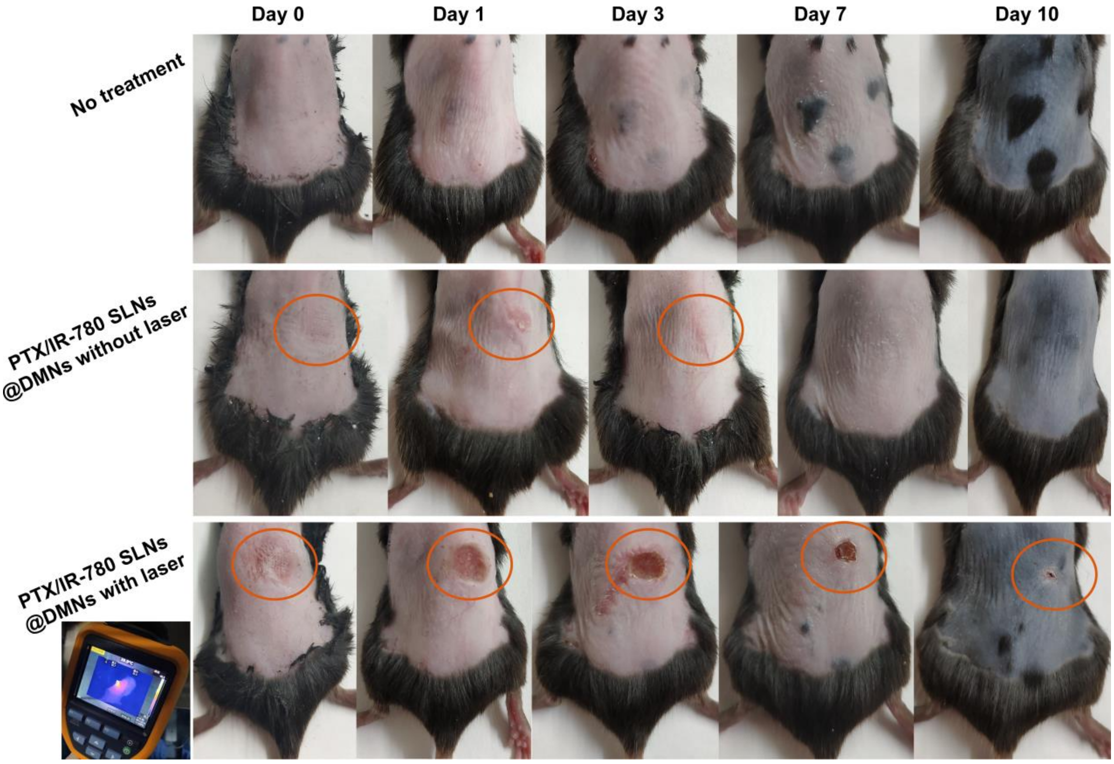

Figure S15. Pictures of mice back skin recovery after treated with PTX/IR-780 SLNs @DMNs with or without laser (808 nm, 1 W/cm^2).

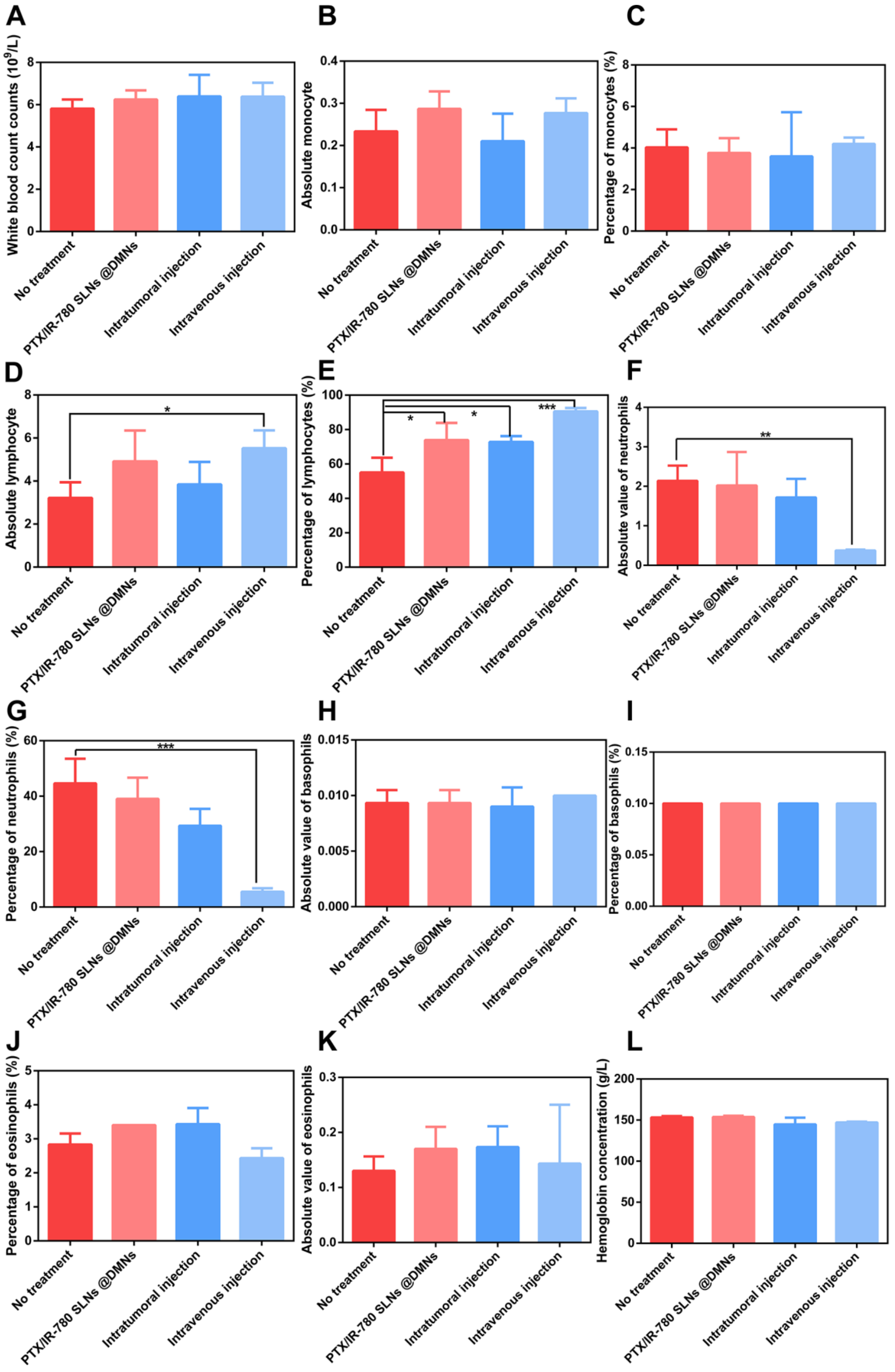
A
White blood count counts (10^9/L)
B
Absolute monocyte
C
Percentage of monocytes (%)
D
Absolute lymphocyte
E
Percentage of lymphocytes (%)
F
Absolute value of neutrophils
G
Percentage of neutrophils (%)
H
Absolute value of basophils
I
Percentage of basophils (%)
J
Percentage of eosinophils (%)
K
Absolute value of eosinophils
L
Hemoglobin concentration (g/L)
No treatment
PTX/IR-780 SLNs @DMNs
Intratumoral injection
Intravenous injection
*
**
***

Figure S16. Changes in blood routine examination parameters in various treatments. Values are expressed as mean ± S.D., *****p < 0.05, ******p < 0.01, *******p < 0.001 versus control (n = 3).

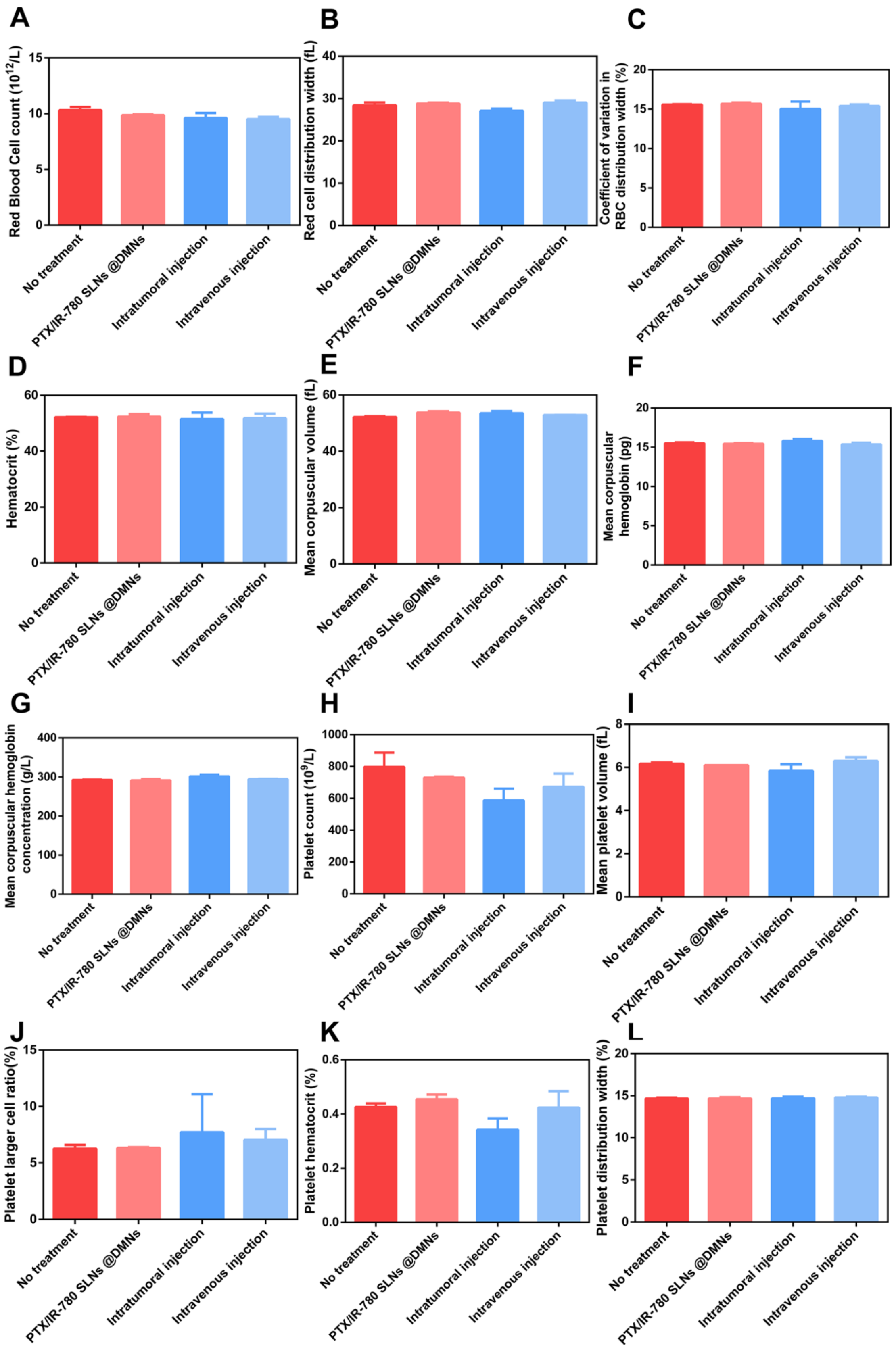

Figure S17. Changes in blood routine examination parameters in various treatments. Values are expressed as mean ± S.D., (n = 3).
